# Current Trends and Challenges for Rapid SMART Diagnostics at Point-of-Site Testing for Marine Toxins

**DOI:** 10.3390/s21072499

**Published:** 2021-04-03

**Authors:** Michael Dillon, Maja A. Zaczek-Moczydlowska, Christine Edwards, Andrew D. Turner, Peter I. Miller, Heather Moore, April McKinney, Linda Lawton, Katrina Campbell

**Affiliations:** 1 Institute for Global Food Security, School of Biological Sciences, Queen’s University Belfast, 19 Chlorine Gardens, Belfast BT9 5DL, UK; mdillonphd@icloud.com (M.D.); mzaczekmoczydlowska01@qub.ac.uk (M.A.Z.-M.); 2 Faculty of Health, Peninsula Medical School, University of Plymouth, Plymouth PL4 8AA, UK; 3 School of Pharmacy and Life Sciences, Robert Gordon University, Aberdeen AB10 7GJ, UK; c.edwards@rgu.ac.uk (C.E.); l.lawton@rgu.ac.uk (L.L.); 4 Centre for Environment, Fisheries and Aquaculture Science, The Nothe, Barrack Road, Weymouth, Dorset DT4 8UB, UK; andrew.turner@cefas.co.uk; 5 Plymouth Marine Laboratory, Remote Sensing Group, Prospect Place, Plymouth PL1 3DH, UK; pim@pml.ac.uk; 6 Agri-Food and Biosciences Institute, 18a Newforge Lane, Belfast, Northern Ireland BT9 5PX, UK; Heather.Moore@afbini.gov.uk (H.M.); April.McKinney@afbini.gov.uk (A.M.)

**Keywords:** marine toxins, methods of analysis, SMART diagnostics, POST, multiplex detection

## Abstract

In the past twenty years marine biotoxin analysis in routine regulatory monitoring has advanced significantly in Europe (EU) and other regions from the use of the mouse bioassay (MBA) towards the high-end analytical techniques such as high-performance liquid chromatography (HPLC) with tandem mass spectrometry (MS). Previously, acceptance of these advanced methods, in progressing away from the MBA, was hindered by a lack of commercial certified analytical standards for method development and validation. This has now been addressed whereby the availability of a wide range of analytical standards from several companies in the EU, North America and Asia has enhanced the development and validation of methods to the required regulatory standards. However, the cost of the high-end analytical equipment, lengthy procedures and the need for qualified personnel to perform analysis can still be a challenge for routine monitoring laboratories. In developing regions, aquaculture production is increasing and alternative inexpensive Sensitive, Measurable, Accurate and Real-Time (SMART) rapid point-of-site testing (POST) methods suitable for novice end users that can be validated and internationally accepted remain an objective for both regulators and the industry. The range of commercial testing kits on the market for marine toxin analysis remains limited and even more so those meeting the requirements for use in regulatory control. Individual assays include enzyme-linked immunosorbent assays (ELISA) and lateral flow membrane-based immunoassays (LFIA) for EU-regulated toxins, such as okadaic acid (OA) and dinophysistoxins (DTXs), saxitoxin (STX) and its analogues and domoic acid (DA) in the form of three separate tests offering varying costs and benefits for the industry. It can be observed from the literature that not only are developments and improvements ongoing for these assays, but there are also novel assays being developed using upcoming state-of-the-art biosensor technology. This review focuses on both currently available methods and recent advances in innovative methods for marine biotoxin testing and the end-user practicalities that need to be observed. Furthermore, it highlights trends that are influencing assay developments such as multiplexing capabilities and rapid POST, indicating potential detection methods that will shape the future market.

## 1. Introduction

**Figure 1:**
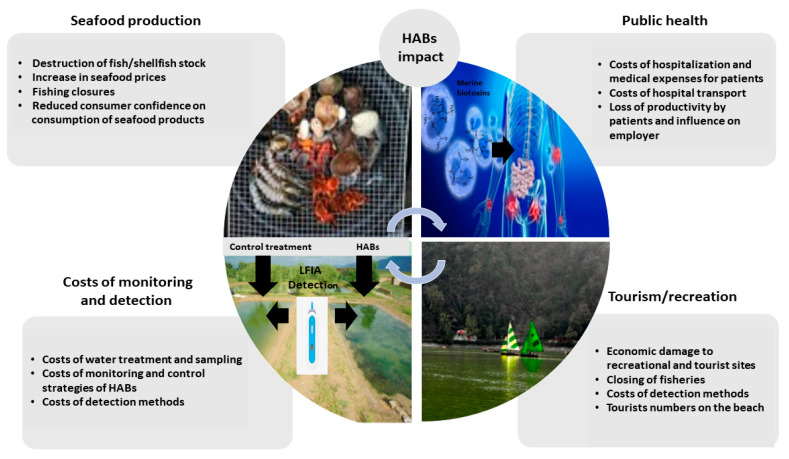
Economic impact of HABs on seafood production, public health, tourism/recreation and costs related to monitoring and detection.

Bivalve mollusc production (e.g., mussels, oysters, scallops, and clams), represents a significant proportion of the seafood industry in Europe and indeed worldwide, estimated to be 17.7 million tons in 2018 [1]. Global exports of molluscs were estimated to be 10.6 billion USD in 2017, with European exports accounting for 20% of global trade. The top European producers are Spain, France, the Netherlands, the United Kingdom, Italy, Portugal and Ireland [2,3]. The production of bivalve molluscs is an important contributor to local economies [1]; however, it is frequently hindered by contamination with marine biotoxins produced naturally by various microalgal species during harmful algal blooms (HABs). HABs typically occur in the warmer months and can have a devastating socio-economic effect and impact on public health [4]. Economic losses caused by HABs can be high, as for example in 2016, the United States seafood industry reported a predicted 900 million USD annual loss due to HABs [5] and are related but not limited to significant delays in shellfish harvesting and commercial sales, the potential destruction or lengthy depuration processes if available of valuable shellfish/fish stock, delayed seeding of new stock, covering of healthcare costs incurred from sick consumers, and in consequence reduced consumer confidence [4] (Figure 1).

In relation to the importance of monitoring biotoxins, millions of dollars are spent each year to prevent HAB events from impacting upon shellfish consumer safety [4]. Therefore, the development of new robust POST methods for the detection of biotoxins is becoming of significant importance especially for food business operators who need a rapid result from harvested shellfish batches.

Shellfish production can be affected by a number of marine biotoxin groups. Table 1 lists these marine toxins along with the harmful phytoplankton species associated with their production.

Phytoplankton sampling for species identification and counting using light microscopy for threshold levels is for most part the precursor for toxin testing in regions of harvest [6]. New approaches to light microscopy using molecular microarrays for determining species have been developed [22,23] as has an electrochemical biosensor for determining toxic algae [24]. Similarly, colour remote sensing methods such as autonomous vehicles and satellite imagery are increasingly being investigated as early warning approaches to predict and determine blooms [25,26] and the use of citizen science smartphone apps are being evaluated to determine water quality [27]. European Commission Regulation No 853/2004 [28] legislates that all shellfish produced must be routinely monitored and tested for the presence of regulated marine toxins before they can reach market. Therefore, although phytoplankton monitoring may allow for informed decisions on bed locations and harvesting both regulators and fishermen require instrumental chemical detection methods to detect toxins to be compliant to the legislation for food safety purposes. This EU legislation focuses on three main groups named originally by their symptoms, specifically amnesic shellfish poisoning (ASP), diarrhetic shellfish poisoning (DSP) and paralytic shellfish poisoning (PSP) as the key regulated groups, as well as additional lipophilic toxins incorporating azaspiracids (AZAs), yessotoxins (YTXs) and pectenotoxins (PTXs) [28,29,30,31,32]. Current legislation dictates that bivalve molluscs placed on the market for human consumption must not exceed the set action limits (Table 1) or the shellfish is deemed not safe for human consumption and the shellfish harvesting areas are closed until toxin levels drop and two successive compliant tests are recorded [32]. These action limits are not exhaustive and do not cover many of the newly emerging toxins, such as cyclic imines (CI), tetrodotoxin (TTX), palytoxin (PLTX) and ciguatoxins (CTX) for shellfish production. However, the legislation states that fish containing paralytic agents or CFP must not be imported into the EU. For several emerging toxins, there is simply not enough data describing the short- and/or long-term health consequences of exposure to these compounds (Table 1) due to the unavailability of analytical standards and lack of toxicological assessment, but as more research is performed, standards become available and more data is generated regarding toxin occurrence and exposure, action limits may potentially be set to include more toxin groups [33]. The European Food Safety Authority (EFSA) opinions on the risk related to tetrodotoxin, palytoxin, brevetoxin, cyclic imines and ciguatoxin consumption have been published. Due to changes in climate and species migration existing, EU regulated toxins are showing up in new regions across the globe whilst currently non-regulated toxins of concern are emerging in new areas and already known toxin hotspots [34,35,36,37,38,39,40]. As such, it is important that shellfish producers are well prepared to screen for additional toxins as and when new regulations are enacted.

Many marine toxin groups have multiple analogues each with varying levels of abundance and toxicity. For example, over 50 analogues of STX have been identified which have been categorized depending on their chemical structures [41,42]. Furthermore, new toxin analogues are periodically being discovered; for example, unique AZA analogues have been identified as recently as 2017 [43,44,45]. Each analogue can be more or less toxic than the parent toxin, therefore, the Panel on Contaminants in the Food Chain (CONTAM) group within EFSA recommends calculating Toxicity Equivalence Factors (TEFs), however, there is still no easy way to do this without in-depth toxicological assessments.

Shellfish producers are often in a difficult position; consumers want freshly caught shellfish, however, with current technologies, it can take between 24 h up to several days from point of sample to return a result to the fisherman. This can be in part dependent on location of the farm relative to the regulatory laboratory but also in part for the time required for regulatory laboratories to process samples and determine the toxin load. By example this delay in turnaround time of analysis caused substantial financial losses for the Australian shellfish industry in 2011 [46]. On occasions the sample for analysis for the harvest may have been taken just prior to a developing HAB at the site and although a result may come back negative, within 24–48 h, a given turnaround time for analysis, the planned harvest can then be contaminated to levels above the maximum permitted level. Following the negative result the produce will be harvested, distributed and eaten whereby thereafter following illness outbreaks, toxin contamination is detected. Similarly, the harvest can be taken at the same time as the official control sample and distribution of shellfish produce underway whereby expensive and reputational damaging recalls are then necessary 24–48 h later following a positive result and consumption of unsafe shellfish. Either scenario can be devastating for both shellfish consumers and producers. Though it should be stated that it is the duty of the food business operator to ensure the food is safe for consumption not the official control laboratory and produce should not be harvested or released until deemed safe. It is also therefore in the best interest of the producer as the food business operator to be able to conduct their own real-time end-product testing to regulatory accepted standards to ascertain if their product is safe to harvest. In relation to these described constraints, recent advances in detection methods for marine biotoxins and improving standards in the food industry can be drivers for change. 

This review outlines and compares the current technologies trialed, applied and commercially available for marine toxin detection and explores potential emerging technologies that may be deemed fit for purpose for producers to screen their harvest before distribution, but also for regulators and stakeholders for monitoring and surveillance along the seafood supply chain.

## 2. Detection Methods for Marine Toxins

**Figure 2:**
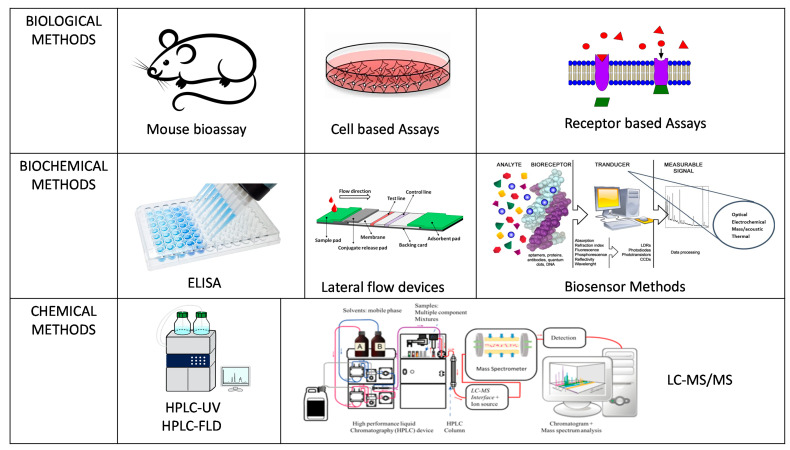
Schematic summary of the various methods of analysis for marine biotoxins.

There are various methods of analysis for marine biotoxins which can be classified into biological, biochemical and chemical based methods (Figure 2) though not all are accepted as official control methods for regulatory purposes and with some acknowledged as proofs of concept for new technological applications. Official control laboratories carry out official testing procedures on behalf of a competent authority following set legislation on accepted methodologies and action limits to stringent standards and validated accreditation. POST as end-product testing procedures are not necessarily following or approved by legislation but can be used by the fisherman for product release as a means to ensure compliance to safety measure for toxin testing for harvest and distribution. The challenge is that the approaches that can be applied for POST end-product testing are often unvalidated and not shown to be fit for purpose for the end user. Therefore, their acceptance for use can be deterred until fully proven in both laboratory and at point of site in their suitability for use. However, it is important to emphasize that the EC Regulation 519/2014 [47] which provided additional information on the validation requirements for semi-quantitative screening methods is driving forward more SMART sensor approaches for approval. The list of currently available end-product testing technologies is provided in Table 2.

## 3. Methods for Official Control Testing

### 3.1. Mouse Bioassay (MBA)

Until recently, for the past 70 years, bioassays such as the MBA have been the reference methods worldwide for identification of DSP [48] and PSPs [49]. MBA methods involve intraperitoneal injection of replicate shellfish extracts to mice and measuring either the time of death after injection, for a direct measure of PSP toxicity, or mortality/morbidity or clinical signs of toxin exposure for DSP. Aside from the obvious ethical issues, testing for the presence of PSP and DSP using the MBA, suffered from low sensitivity, inaccuracies due to matrix interferences, whilst offering no information regarding the toxin profile present in the shellfish extract or the toxin(s) responsible for death. Following implementation of EU Directive 2010/63 (on the protection of animals used for scientific purposes) which prohibits use of an animal test when an accepted alternative exists [50], MBA have been gradually replaced in recent years in the EU (i.e., in UK and Ireland MBA is not in use) with alternative non-animal methods for shellfish toxin detection. Moreover, as a result of the scientific, technical, and ethical limitations, countries such as Australia, Canada and New Zealand no longer use the MBA test for routine toxicity testing of shellfish. However, this progress in moving away from the MBA was not without its challenges in method development, validation and translating toxicity to the MBA, with equivalency factors [41,51]. Following implementation of Commission Regulation (EU) 2017/1980, the MBA is no longer the reference method for detecting PSP toxins in the EU [32] and is not listed in the National Shellfish Sanitation Program (NSSP) ‘Guide for the Control of Molluscan Shellfish’ as a method to test for DSP or ASP. This change should ensure the complete replacement of the MBA at the EU level.

### 3.2. Chemical Methods

In 1997, the Council of the European Union adopted Directive 97/61/EC (Council of the European Union, 1997) [52], allowing liquid chromatography (LC) with UV detection (LC-UV) to be used to screen for DA as the main ASP toxin [53]. In 2011, the European Commission adopted Regulation (EU) No 15/2011 (European Commission, 2011) [31] to approve LC with tandem mass spectrometry (LC-MS/MS) for the screening for lipophilic toxins, including DSP [54]. More recently, the European Commission adopted Regulation (EU) No 2017/1980 [32], accepting the so-called Lawrence method (AOAC 2005.06 official method of analysis) as the EU reference method for PSP detection. The Lawrence method involves liquid chromatography (LC) with pre-column oxidation and fluorescence detection [55,56]. Alternatively, LC with post-column oxidation and fluorescence detection has been approved for PSP detection in the United States and Canada [57].

The technique of electrospray ionization (ESI) liquid chromatography-mass spectrometry (LC-MS) has proven to be one of the most powerful tools for the detection, identification and quantification of marine toxins. Suzuki et al. [58] recently conducted a review on LC-MS analysis of marine toxins which concluded that similar MS/MS fragmentation patterns obtained by different equipment demonstrate that MS/MS spectra of marine toxins could be useful fingerprints for identification of toxins. Further improvements in these methods are always being considered with new combined all toxin detection methods being developed and validated for use. Rodríguez et al. [59] recently developed an ultra performance liquid chromatography (UPLC)-MS/MS method for the identification and quantification of hydrophilic and lipophilic marine toxins. The method included the determination of 14 toxins of the STX group, 15 lipophilic toxins, 15 toxins of the TTX group and DA. Validation studies demonstrated acceptable method performance characteristics for linearity, and repeatability between-batch and within-batch. The study demonstrated that the UPLC—MS/MS method provides a potentially useful tool to determine hydrophilic and lipophilic toxins and therefore it could be appropriate for interlaboratory validation. 

Chemical detection methods incorporating chromatographic separation and a range of detectors are now the current diagnostic gold standards for marine toxin detection: they are accurate, sensitive, and provide information on the specific toxins for whichever standards are commercially available. Similarly, sample preparation methods can be complex whereby after solvent extraction, the crude shellfish extract may be submitted to solid-phase extraction (SPE). Clean-up steps are usually employed to either pre-concentrate extracts to enhance assay sensitivity, or to remove matrix co-extractives which interfere with the analysis. SPE involves dissolving or mixing the analyte (s) of interest in a liquid sample (mobile phase) and then passing that sample over a solid chromatographic component (solid phase). The desired analytes will either flow through the solid element, remaining in the mobile phase, or be retained on the solid phase and subsequently eluted for testing. SPE clean-up is critical for the liquid chromatography fluorescent detection (LC-FLD) or LC-MS/MS analysis of PSP toxins [55,60] and can be used for reducing matrix interferences during ASP analysis [61]. These et al. [62] have used SPE to enrich OA, AZA, PTX2, and YTX up to a factor of 10, increasing the sensitivity to low level toxins and reducing the amount of sample needed for testing. Gerssen et al. [63] performed SPE using polymeric sorbents, coupled with LC under alkaline conditions, to effectively reduce matrix effects with methanolic extracted hydrophilic marine toxins when measured by LC-MS/MS [63]. For regulatory high throughput monitoring of lipophilic toxins by LC-MS/MS, however, SPE is not typically applied. That said though, they are also specialized and expensive procedures: they require trained staff, a large range of disposable reagents, bulky and expensive laboratory equipment, and dedicated laboratory facilities. Therefore, they are limited to the regulatory or large commercial laboratory settings, and as such, they are not available to producers for rapid, on-site testing during harvesting. However, with the immediate challenge in replacing the mouse bioassay as the driver for innovation in alternative methods now achieved from a regulatory perspective, the current diagnostic gold standard LC and LC-MS/MS based methods are now established as a benchmark to compare other tests. 

### 3.3. Receptor Binding Assay (RBA)

Functional assays such as receptor binding assays were also examined and utilized to move away from the use of animal testing for toxicity determination [64]. The RBA relies on the specific recognition of an analyte via specific receptors which are often endogenous targets of the analyte. Doucette et al. [65] described a microtiter plate-based RBA for PSP testing of bivalve mollusc species where a STX standard or sample is added to a 96-well plate, followed by tracer-tagged STX, and lastly diluted rat membrane homogenate. The membrane homogenate is derived from rat brains and therefore contains the biological target of STX, voltage-gated sodium channels on nerve cells. The STX or analogue binding to the sodium channel is dependent on the toxin’s affinity to the endogenous target, and because toxin’s affinity for biological receptors is a direct reflection of its toxicity, the RBA uniquely gives an indication of the toxicity of a sample, as well as the amount of toxin present. This RBA has been successfully validated in both single-laboratory [66] and in multi-laboratory [67] studies for PSP detection. This assay is currently validated for screening of mussels and clams for PSP toxins in the US and studies have demonstrated performance characteristics for oysters, mussels and clams in Europe [68]. Such tests have been implemented in aquaculture regions of the developing world as opposed to Europe as health and safety measures in Europe for the use of radioactive materials causes restrictions in its use. 

Alonso et al. have taken the determination of toxicity one step further by employing automated patch clamp sensors [69]. Automated patch clamp sensors can assess action potential propagation along neurons and have therefore been used to quantify the functional inhibition of neuronal signaling by 9 different PSP analogues. Furthermore, Alonso et al. [69] used mouse cell lines transfected to express various human sodium receptor subtypes. This has the great advantage of avoiding the risk of species-dependent differences in toxin susceptibility.

DSPs are potent protein phosphatase 2A (PP2A) inhibitors and thus very well suited to the RBA format [70]. Indeed, Zeulab produce the OkaTest kit, a commercially available DSP RBA that is also currently validated as a supplementary test to the reference screening method [71]. The OkaTest kit comes with PP2A and a PP2A substrate (p-Nitrophenyl phosphate). It is a colorimetric assay and it takes 1 h to set up, and 30 min to run. It has been validated in molluscs in both single [72] and multi-laboratory [73] studies for DSPs detection, and therefore can be used as a supplementary test to the reference method for determination of DSPs according to the Commission Regulations (EC) No. 853/2004 and No. 15/2011 [28,31].

RBAs are also in development for other toxins. For example, Aráoz et al. have designed an RBA capable of detecting for anatoxins (ANTX) in drinking water utilizing nicotinic acetylcholine receptors (nAChRs) [74,75]; ANTX is a potent agonist of nAChRs. Instead of rat brain homogenate, this assay uses Torpedo electrolyte membranes, as these membranes are rich in nAChRs. Fonfría et al. have further developed this method to detect GYM and SPX in the nM range [76]; Rodríguez et al. subsequently optimized the assay for high-throughput [77]. Furthermore, Hardison et al. have developed a similar RBA to detect CTX from reef fish tissue. Their assay can sufficiently quantify CTX well below the limit set by the USFDA. Additionally, their CTX RBA is reported to correlate well with other methods [78]. 

Pelin et al. [79] have taken a different approach in their PLTX assay. Instead of competitive-based inhibition, their assay was designed as a sandwich; PLTX is captured by receptors on immobilized HaCaT cells and then detected with labelled antibodies. This assay is more sensitive (32 pg/mL), more specific, and significantly faster than other PLTX immunoassays, and it has been validated by inter-laboratory studies in mussel tissue samples. 

RBAs can be an accurate and sensitive means of measuring toxicity in shellfish samples, but as with the MBA does not return information on individual toxins present, preventing determination of toxin profiles. Additionally, other contaminants can act on these receptors whereby specificity of the test to one toxin family or other chemical contaminants perhaps also present cannot be determined and competing interfering compounds can present misleading results. Also, specialist equipment and reagents are required and the inclusion of the receptors as resources from the use of animals remains to have ethical implications, all of which preclude the uptake of these assays and their application on-site for the producers. 

## 4. Methods for End-Product Testing (EPT)

### 4.1. Immunoassays

#### 4.1.1. Enzyme-Linked Immunosorbent Assays (ELISA)

**Figure 3:**
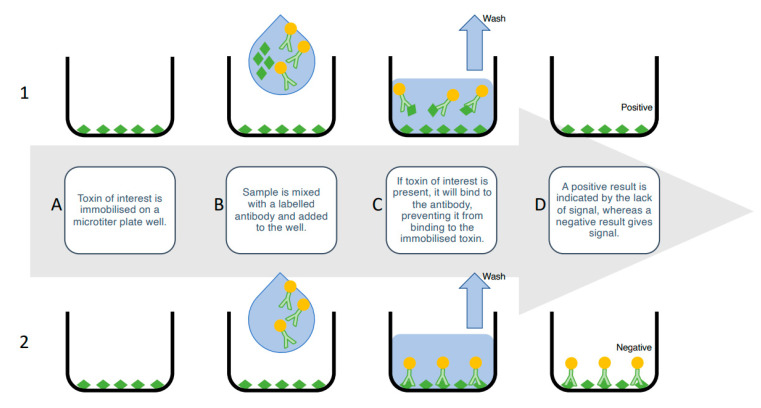
Inhibition-based competitive enzyme-linked immunosorbent assay. (**A**) The toxin of interest is immobilized in the solid phase on a microtiter plate well. (**B**) Sample is mixed with a labelled antibody and added to the well. (**C1**) If the toxin of interest is present in the sample, it will compete with the immobilized toxin to bind to the labelled antibody. (**C2**) If the toxin of interest is not present, the labelled antibody will bind to the immobilized toxin. (**D**) Unbound antibody is washed out and the wells imaged. (**D1**) A positive result is indicated by the lack of signal, because toxin in the sample has prevented the antibody from binding, whereas (**D2**) a negative result is indicated by a signal, because the labelled antibody has not been inhibited from binding to the immobilized toxin.

ELISAs rely on the highly specific and sensitive nature of antibodies to detect the specific toxin structures and quantify the toxin concentrations in a solution. There are a number of ELISA variations, including direct and indirect, however, the competitive-indirect ELISA is the most popular for shellfish toxin screening [80,81]. In this format, an analytical standard of toxin is immobilized on a 96-well microtiter plate. Next, the sample is added, followed by the primary antibody, which is specific to the toxin. Next, a secondary, enzyme-conjugated antibody, which recognizes the primary antibody, is added, followed by an enzymatic solution that reacts with the enzyme-conjugated secondary antibody. A colorimetric readout is given; this is inversely proportional to the amount of toxin present in the sample; toxins in a contaminated sample will compete with the plate-bound toxin for the primary antibody. A weaker colour indicates less primary antibody binding to the plate-bound toxin, because there is more toxin in the sample blocking binding (Figure 3).

In the early days of ELISA development, Garthwaite et al. produced a number of ELISAs capable of detecting DA, OA, STX, BTX, and YTX and demonstrated the feasibility of an ELISA-based screening system for identifying contaminated shellfish [82]. Currently, there are multiple options for commercial ELISA kits available for use in end-product testing (Table 3). These include tests by Bioo Scientific, Zeulab, Creative Diagnostics, Mercury Science, Abraxis, Marbionc, Unibiotest, Beacon, R-Biopharm, and Biosense Laboratories. A summary of these is outlined in Table 3, and more details about some of these can be found in McLeod et al.’s review [83].

Although ELISA technology is now substantially dated compared to novel biosensor approaches it is still the most highly utilized rapid screening assay in food and clinical diagnostics worldwide due in part to the expense and complexities in acceptability of other methods. 

Due to the wide range of analogues of high potency and varying toxicities the paralytic shellfish toxins were one of the most challenging toxin groups for ELISA development. The first antibodies were developed to saxitoxin (STX) in 1964 by Johnson et al. [84] and the first ELISAs only utilized antibodies raised against STX. However, due to the highly specific nature of antibodies, these tests were not able to accurately assess total PST levels in a sample. For example, neosaxitoxin (NEO), the second most toxic STX congener, would often be underreported in these assays [85,86,87], and as such, multiple assays had to be run side-by-side to accurately assess total PSP levels [88]. 

To simplify this analysis, Huang et al. [89] used two antibodies within a single heterologous ELISA to produce an assay capable of detecting both STX and NEO at the same time. By combining two antibodies, Huang et al. [89] were able to increase the sensitivity and specificity of their test to detect total PSP levels similar to the MBA. McCall et al. [90] have developed a similar ELISA capable of detecting STX-analogues using a single monoclonal antibody (mAb) that recognizes STX and NEO, but does not cross-react very well with GTX1/4 nor GTX2/3. In this assay, GTX must be converted to STX by incubating the sample with L-cysteine. A number of other STX ELISA kits have been developed, as reviewed [83]. Details regarding the cross-reactivities of the test response are particularly important given the wide variability in PST analogues present in different geographical regions [91] and their varying toxic potency. Cross reactivities are known to vary significantly between different commercial ELISA kits. Five ELISAs were compared in terms of their qualitative and quantitative performance, with ELISA results assessed against the regulatory LC-FLD method. Performance varied considerably between the commercial assays, specifically with some under-estimating and others over-estimating toxicity due to cross-reactivity related issues particularly relating to the presence of PST analogues such as GTX1/4 and linearity problems [91].

For DSP testing of the okadaic acid (OA) group of toxins, including OA and the Dinophysis (DTX) toxins, many ELISA kits also suffer from cross-reactivity issues [92,93,94,95,96]. Anti-OA antibodies can have limited cross-reactivity with DTXs, especially DTX-2 [97]. Given the potential for a high proportion of OA-group toxins to be present in shellfish as acyl-esters, ELISAs which do not incorporate an alkaline hydrolysis step to convert esters into freely-occurring OA and DTXs will potentially significantly under-estimate total OA-group toxicity [98]. Various studies have assessed the performance of commercial DSP ELISAs, in comparison with the LC-MS/MS DSP regulatory method. Differences in performance were reported, with most tests exhibiting issues relating to low cross-reactivity to DTX2 [99,100,101].

As the simplest of the EU regulated toxin groups in relation to the single congener domoic acid to be reported there have been numerous ELISA kits developed for ASP detection [81,102,103,104,105,106,107,108,109,110,111]. Biosense currently has the only commercial ELISA kit for ASP detection that has been validated in single [112] and multi-laboratory [113] studies to AOAC standards. The kit is based on antibodies developed by Garthwaite, et al. and has a limit of quantitation of 38 ng/g flesh [102]. Uniquely, Shaw et al. isolated recombinant sheep single-chain antibody fragments (scFvs) to create a novel ELISA [114]. This assay was tested with naturally contaminated scallop tissue and correlates well with standard high-performance liquid chromatography (HPLC) assay results, although the ELISA tended to underestimate DA content. Johnson et al. compared the performance of three ELISA kits for DA detection and quantitation, with all providing an acceptable level of qualitative detection, but with variable quantitative performance [99].

Ling et al. developed an ELISA capable of detecting BTX at levels as low as 14 ng/mL [115]. This assay was tested with a wide range of artificially spiked shellfish samples, including clams, mussels, oysters, and scallops and found to have an average toxin recovery rate of 89 ± 2%. Unfortunately, naturally contaminated shellfish samples were not tested. Briggs et al. used sheep polyclonal antibodies (pAbs) to develop an ELISA capable of detecting YTX and a broad selection of YTX analogues. This assay reports higher YTX levels in mussel flesh compared to LC-MS, however, this could be because there are analogues present in the extract that the ELISA detects but the LC-MS does not [116].

Samdal et al. have developed a competitive ELISA capable of detecting AZA and analogues in shellfish samples and algal cells. It has a quantitative range of 0.45–8.6 ng/mL and limit of quantitation (LOQ) of 57 µg/kg in shellfish, satisfying the AZA maximum permitted level (MPL) of 160 µg/kg [117]. Reverté et al. have developed a maleimide-based ELISA (mELISA) capable of detecting TTX in pufferfish. In this assay, TTX is immobilised on maleimide-activated dithiol-carboxylate monolayers; this strategy results in more structured antigen immobilisation and increases assay fidelity [118]. This assay was further refined for use in oysters and mussels [119], and both assays are capable of detecting TTX well below 2 μg/g of flesh, the permissible limit set by the Japanese government for puffer fish.

Tsumuraya et al. have developed a sandwich ELISA whereby CTX is captured between immobilised and labelled antibodies. Their test can detect four Pacific CTX (P-CTX) congeners at concentrations as low as 1 pg/mL [120]. The European Commission does not currently have an MRL set for CTX, and thus official, regular testing does not occur, however, the EU Community Reference Laboratory on Marine Biotoxins (CRLMB) has recommended a guidance level of 40 ng/kg flesh [121]. On the other hand, the United States Food and Drug Administration has established guidance levels of 10 ng/kg flesh of Caribbean CTXs (C-CTX) and 100 ng/kg flesh of Pacific CTXs (P-CTX), and uses a two-tiered testing protocol involving (1) a mouse neuroblastoma (N2a) cell assay and (2) an LC-MS/MS assay [122,123]. 

Boscolo et al. have developed a sandwich ELISA capable of detecting PLTX and 42-OH-PLTX in natural samples as low as 1 ng/mL, however, unfortunately the assay could not be assessed with other PLTX analogues [124]. The EFSA advises an MRL of 30 ug/kg flesh, however, there is currently no legislation requiring PLTX testing in Europe. 

There are a large number of ELISA tests developed and commercially available for regulated toxins. There are also many tests developed for non-regulated/emerging toxins. Even still, ELISA is not a widely accepted screening method for all marine toxins, in part due to the sensitivity and specificity of a test relative to the congeners in a toxin group and the lack of available toxin standards to calibrate the method. Shellfish toxins from some classes can be highly lethal in small doses, and thus some are listed as controlled substances, most notably STX and analogues. This limits their availability as reference reagents in competitive-based assay formats and greatly increases production costs of these types of test. Additionally, ELISAs can be time consuming procedures with many steps whereby the risk for human/operator errors increases. They require availability of laboratory instrumentation such as plate readers, incubators and shakers, and protocols for some of the assays are not as easy to follow as some other end product tests [91,99]. Although there are a significant number of ELISA applications commercially available the restrictions in demonstrating the methodology to be fit for purpose in single laboratory and interlaboratory validation remains an essential requirement in legislation for their uptake for regulatory purposes. The time and cost of conducting these studies by a commercial entity to regulated standards, such as AOAC validation protocols, therefore has to be viable for the business entity in terms of potential sales value thereafter whereby due to the limited size of the market for sales there can be a catch 22 in uptake due to lack of validation for regulatory purposes. Therefore, with few exceptions these tests are deemed either as a research tool or if considered as a POST for end product testing they are only applied for use as a screening tool by the food business operators at their own risk with limited data available on their validity.

#### 4.1.2. Lateral Flow Immunoassay (LFIA)

The LFIA has recently become a very popular assay format for all kinds of diagnostic testing as a single analysis portable tool though the portability or novice end user application of such a device can be restricted with the sample preparation required prior to usage of the device. Like the ELISA, the LFIA utilizes specific antibodies to recognize toxins in a sample. There are also several types of LFIAs; typically, shellfish screening is done with a competitive-based assay. An LFIA comprises a series of overlapping membranes that function based on capillary flow. At one end of the test, a conjugated antibody is sprayed onto a sample pad whilst on the other end of the test, toxin is immobilized on a membrane (e.g., nitrocellulose) at the test line. Liquid sample is applied to the sample pad, where the conjugated antibody will interact with any toxin in the sample. Capillary action then pulls the complex along to the test line where any free conjugated antibody will bind to the toxin. Like the competitive ELISA, the amount of binding is inversely proportional to the amount of toxin present in the sample. The antibody may be conjugated with any number of signal molecules, including colloidal gold particles, nanorods, chemiluminescence, or fluorescence.

Jawaid et al. have developed a qualitative LFIA, utilizing colloidal gold nanoparticles for detection of PSTs in scallops, oysters, clams, mussels, and cockles. The test uses a cocktail of antibodies raised against different paralytic shellfish toxins [125]. These antibodies demonstrate broad cross-reactivity with 128.9% binding to NEO, 23.4% to GTX2/3 and 55.6% of dcSTX, amongst others (relative to 100% STX binding). This assay takes 15–20 min to prepare the samples, 5 min to complete the assay, and it was validated in single-laboratory and multi-laboratory studies to AOAC standards, although there is noted variability in mussel samples [46,126].

The same group has produced a similar assay for the assessment of ASP and DSPs [127,128]. The DSP detection kit can detect OA-group toxins in scallops, clams, and mussels [101] as well as oysters [91,100]. Neogen advertises an LOD of 160 µg/kg, although values as low as 60 µg/kg can test positive [128]. This can be frustrating for producers when their harvest is well below the action limit, but still testing positive by LFIA. The assay also includes the alkaline hydrolysis step to liberate esterified OA-group toxins. Scotia also produces a range of LFIAs for marine shellfish toxin detection, including DSP, ASP and PSP test kits [99,129,130]. The Scotia PSP LFIA uniquely incorporates an additional conversion step, to transform GTX1/4 and GTX2/3 toxins, with the former in particular exhibiting very low cross reactivities, to NEO and STX respectively, thereby decreasing the occurrence of false negative results [131]. The Neogen and Scotia kits are qualitative, and the purchase of an optional test strip reader is advised. However, these readers are also able to provide test result numbers which can be used to provide a semi-quantitative indication of the levels of toxicity present [91,131] though this component is not recommended by the kit manufacturer for end user use. A summary of commercially available LFIAs is outlined in Table 2, and more details about some of these can be found in McLeod et al.’s review [83]. 

Other groups have also developed prototype LFIAs for TTX and BTX. Uniquely, Shen et al. used quantum dots and gold nanoflowers to create an LFIA that could detect TTX concentrations as low as 0.2 ng/mL [132]. Ling et al. adapted their BTX ELISA (discussed above) into an LFIA, however, unfortunately, the new LFIA format had significantly reduced sensitivity (200 ng/mL) compared to the ELISA format (14 ng/mL) [115].

The LFIA format is robust, stable, and rapid (15 min). Also, it requires very little technical expertise or experience. As such, it is a good candidate for on-site analysis. Unfortunately, the current commercially available LFIAs are qualitative and the competitive assay format requires a test strip reader to confidently read the results. Whilst a ‘yes’ or ‘no’ result can be simple to understand, more nuanced data can be helpful. For example, if toxin is detected, an LFIA cannot determine how much is present, other than the semi-quantitative indications provided by the automated scanners. Similarly, it can be difficult to determine trends with an LFIA, such as, if a contamination incident is getting better or worse over time on a positive or negative classification of result only. Similar to the ELISA methods there are a number of commercial LFDs available but the same limitations apply in their validation for regulatory purposes even as a screening tool. Performance criteria for the validation of qualitative and semi-quantitative screening methods for certain mycotoxins has been set in European Regulation (EC) 519/2014 whereby these could also translate to methods for the screening of marine and freshwater toxins [47]. Demonstrating the methods to be fit for purpose to accredited standards would improve their uptake for use and commercial viability but as already stated there is a cost benefit analysis for the diagnostics industry in doing so which currently prevents this. Researchers are therefore conducting such studies to provide and enable timely solutions for the shellfish industry and regulators for enhanced safety performance [46].

## 5. Proof of Concept Biosensors

A number of prototype and commercial single and multiplex biosensors have been evaluated for the purpose of marine toxins determination though steps from innovation to the commercialization of these technologies for use in the laboratory or in the field has not been forthcoming to date [133]. Similarly, several biorecognition molecules such as antibodies, aptamers and other receptors for certain toxins detection have been evaluated to be used on different handheld devices in their ability to be fit for this purpose. Among the use of aptamers or other receptors, the application of antibodies as biorecognition molecules was the most investigated for marine biotoxins detection on a wide range of sensor platforms due to their specificity and sensitivity. Nonetheless, there are still obstacles to reach the stage of commercialization of these sensing platforms, and similar to ELISA or LFD development, the lack of full validation to the regulatory requirements for their uptake in marine toxins detection shelves these technologies as a proof-of-concepts or research tools at universities and research institutes. 

### 5.1. Antibody-Based Biosensors 

#### 5.1.1. Flow-Through Microarrays

An alternative screening method developed by Szkola et al. exploits an automated flow-through chemiluminescence microarray for simultaneously detecting DA, OA, and STX in shellfish. Toxins are ‘spotted’ onto a polyethylene glycol-treated glass slide and a mixture of the primary antibody and sample is added. Next, a horseradish peroxidase-conjugated secondary antibody is added. The secondary antibody binds to the primary antibody to produce a chemiluminescent signal [134]. 

This assay requires 15 min of sample preparation and takes 20 min to complete. The microarray achieved a LOD of 0.4, 0.5 and 1 µg/L and recovery values of 61.6%, 86.2% and 102.5% for STX, DA and OA respectively in spiked shellfish samples. (Cross-reactivities were not assessed for toxin analogues.) Furthermore, the readouts for this multiplex were quantitative for OA and DA, but only semi-quantitative for STX. Notably, the microarrays may be regenerated with the addition of an SDS-HCl buffer after the assay which allows them to be re-used for a total of 25 measurements. Whilst rapid, relatively simple-to-use, and offering multiplexing capabilities, the equipment required is too sophisticated and cumbersome to be used in the field; it is about the size of a standard fume hood. Additionally, glass slides are fragile and can be easily broken. Furthermore, similar to the immunoassays described above, this is a competitive-based inhibition assay and thus it suffers from the same drawbacks (qualitative).

#### 5.1.2. Fluorometric Assays

Fraga et al. have developed a multi-detection, semi-quantitative immunoassay, using a solid-phase microsphere-flow cytometry system based on the Luminex xMAP^®®^ technology. This assay is capable of detecting OA, DA and STX in mussels and scallops [135]. It is also a competitive-based inhibition assay. The three toxins are conjugated to Fluorophore-encoded Luminex microspheres, which are then mixed with sample, anti-toxin primary antibodies, and reporter secondary antibodies. It is similar to the previous cited assays, binding of the primary (and thus secondary) antibodies to the toxin-conjugated microspheres is dependent on toxin levels present in the sample. High levels of sample toxin reduce primary antibody binding to the microspheres, and thus result in a lower signal when read by a Luminex 200 analyser. Using this approach, the authors could screen up to 40 samples per run, however, the runs themselves are not particularly rapid, owing to the multiple 1-h incubation periods required for each group of samples during the preparation phase. Also, similar to the fluorometric assay described above, Luminex technology is large, expensive (particularly for the magnetic particles), and not suited to on-site testing.

Of note, the authors used a methanol/acetate buffer for solvent extraction and were able to obtain recoveries of 80%, 90% and almost 100% for OA, STX, and DA respectively. Inhibitory Concentration 50 (IC_50_) values were also reported: 5.58 ng/mL for STX, 1.15 ng/mL for OA, and 1.92 ng/mL for DA. To date, this assay has not been assessed for cross-reactivity to any of the various toxin analogues or other toxin groups. 

This technology was also evaluated further for the detection of azaspiracid [136] using a monoclonal antibody whereby applying the simple acetate/methanol or methanol extractions yielded final extracts with no matrix interferences and adequate recovery rates of 86.5% and 75.8%, respectively. Though limitations in its acceptability arise from the lack of knowledge of cross-reactivity of the test with all azaspiracid congeners that can now be determined by LC-MS/MS in real samples. 

On the same note the application of using the technology for the determination of cyclic imines using a receptor based assay has also been shown [137]. 

#### 5.1.3. Surface Plasmon Resonance (SPR)

Since 2000, SPR biosensors have been employed for the detection of toxin analytes in a variety of sample types [138,139,140,141,142,143,144,145,146]. A surface plasmon polariton is an electromagnetic wave that runs parallel along a metal surface at its interface with the surrounding air or other medium. The oscillations of those electromagnetic waves are highly sensitive to changes to that metal surface, for example through binding of different molecules. When light is shone through a prism, the angle of its refraction upon contact with the metal surface is dictated by the oscillations of the surface plasmon polaritons. A sensor then measures the amount of light that is reflected towards a given point which gives an indirect measurement of how much material has bound to the metal surface. Many SPR biosensors use immune recognition and, much like other immunoassays, they can be designed as direct, indirect, or inhibition-based competition assays. Due to the highly sensitive nature of SPR, it is imperative that there is no non-specific binding of molecules to the metal surface which will influence the readout. 

Yu et al. describe an inhibition-based competitive SPR assay for the detection of DA [147]. DA is immobilized on mixed self-assembled monolayers (SAMs) on a gold-coated sensor chip. The sample is incubated with anti-DA antibody, introduced to the sensor chip, and optical sensorgrams recorded; unbound antibodies interact with the immobilized DA to produce a change in the amount of reflected light and thus indirectly indicate the amount of toxin in the sample. The chips are reusable and can be regenerated with sodium hydroxide solution. The assay is highly sensitive, with a LOD of 0.1 ng/mL, although it is important to note that this is with DA prepared in buffer and not in shellfish sample. 

Campbell et al. showed the proof of principle of PSP detection utilizing different antibody and receptor binders [138] whereby this was followed up by Fonfría et al. in the development of the inhibition-based competitive SPR assay to measure PSP toxins in mussels, oysters, cockles, scallops and clams [148]. Anti-GTX2/3 antibodies were used in conjunction with a STX-carboxymethylated dextran, gold-coated sensor chip. The assay was able to quantify STX and GTX2/3, as well as a number of their analogues, ranging from 2 to 50 ng/mL. Unfortunately, the SPR biosensor gave a 5-fold higher measurement of the toxins for some samples compared to the MBA and HPLC, showing poor correlation with reference methods. This was due to the antibody having a high affinity for the less potent toxins GTX5 and C1/C2. More recently, the method was refined by Campbell et al. using an anti-STX antibody which resulted in improved correlation with data obtained from HPLC (92% agreement) and MBA (96% agreement) methods, taking 6 min to run, and accommodating up to 40 samples per hour [141]. Furthermore, it has an LOD of 120 µg/kg for STX in mussels and has been validated in single-laboratory studies to accredited standards [141] and proven in principle within a pilot interlaboratory study [145].

SPR assays have also been used for the detection of DA in clams [149] OA in mussels [150], and for the multiplexing of PSP toxins, OA, and DA in algal and seawater samples [151] and shellfish matrices as a move towards the optoelectronic mouse [152]. 

There has been little work with SPR for emerging toxins. Yakes et al. developed an SPR assay to detect PLTX in grouper and clams [146]. This assay can detect sub-ng levels of PLTX, well below the EFSA proposed limit. Campbell et al. have developed and validated an SPR assay to detect TTX [140]. This assay can detect TTX levels as low as 200 μg/kg and each chip is capable of running over 800 samples in total. The assay was validated in single-laboratory studies to AOAC standards for gastropods and puffer fish and can detect TTX levels 10-times lower than the permissible limit set by the Japanese government. Most recently, a field applicable SPR biosensor based on antibody inhibition assay to detect DA in the seawater was reported as to be highly sensitive tool. This sensor was able to detect DA at low concentrations (0.1–2 ng/mL). However, this prototype has certain limitations i.e multiplexing the assay increasing of the detection range and reproducibility [144]. SPR assays have a lot of advantages over other, more traditional methods. They are label-free, rapid, automated, require small amounts of sample, and provide real-time measurements. Furthermore, sensor chips are reusable and can be multiplexed to detect multiple toxins in a single run. Unfortunately, however, SPR was initially dominated by Biacore SPR systems and on acquisition with GE Healthcare the food sector was no longer seen as a lucrative market for the use of the technology due to the initial investment in expense of the technology required with only large multinational organizations able to afford the technology for food safety analysis. Furthermore, the equipment requires regular maintenance, and is not particularly portable for in situ analysis for this application as requested by producers. For this reason research in this area wound down and other technologies were explored for enhanced portability. There was some work to develop this technique in a miniature version [149], however, this technology still requires extensive validation and has not shown its initial promise in this field for commercialization.

Nonetheless, SPR remains a useful tool for antibody selection for these toxins and in assessing single antibodies, cocktails of antibodies or bispecific antibodies for selectivity and specificity towards toxin mixtures relative to their total toxicity in the mouse bioassay [139]. 

#### 5.1.4. Electrochemical Biosensors 

Electrochemical biosensors can analyse the contents of a biological sample and convert biological information into an electronic signal that can be easily processed and interpreted. Electrochemical assays can be highly sensitive, and their implementation is versatile. Bratakou et al. describe a STX immunosensor, which incorporates a STX antibody on a lipid bilayer, that is then added to a graphene nanosheet electrode. A silver reference electrode allows a potentiometric electrochemical measurement to be detected when toxin binds [153]. The sensor was tested with mussels and oysters, has a run time of up to 20 min, and can be regenerated for re-use. 

Leonardo et al. developed a competitive-based electrochemical immunosensor aimed at detecting AZA and analogues [154]. Anti-AZA antibodies are immobilised to protein G or avidin-coated electrodes on screen-printed carbon 8-electrode arrays. The sample is premixed with HRP-conjugated AZA-1 and added to the sensor; free AZAs compete with the conjugated AZA-1 to bind to the immobilised antibody. HRP substrate reduction gives an electrochemical signal that is inversely proportional to the amount of free AZA in the sample. The assay achieves a broad dynamic range capable of detecting AZA and numerous analogues below the MRL of 160 µg/kg, including AZAs 1–10 and potentially AZA carboxy congeners. Whilst the detection capabilities of protein G and avidin-immobilised anti-AZA antibody were similar, when a regeneration step was added, the avidin-bound anti-AZA antibody survived to a much higher degree, allowing sensor re-use up to 6 times. Furthermore, when the assay was miniaturised, the quantities of AZA antibody was significantly reduced; this is beneficial given the low availability of AZA toxin for antibody development. 

Zamolo, et al. describe a different approach, a novel hybrid system combining a sandwich immunoassay and electrochemiluminescence (ECL) to detect PLTX [155]. In this assay, measured ECL is directly proportional to PTX concentration, and PTX can be detected in both mussel and algal samples at concentrations as low as 220 ng/mL. 

#### 5.1.5. Planar Waveguide Cartridges

McNamee et al. applied an MBio cartridge to simultaneously identify five different marine and freshwater toxins from a single sample [156]. The cartridge combines planar waveguide with fluorescence and functions similar to an LFIA as described above: it is a competition-based assay whereby toxin conjugates are spotted onto a plastic slide, making the signal inversely proportional to the amount of toxin present in the sample. Uniquely, this sensor uses fluorescently labelled reporter antibodies. Fluorescent reporters can be more sensitive than traditional gold reporters; thus, it takes less sample to generate a positive result. The test zone can be smaller, in this case, a spot instead of a line, and you can multiplex more tests on a single cartridge. 

The test developed by McNamee et al. can detect up to five different marine and freshwater toxins on a single cartridge: STX, DA, OA, microcystin-LR and analogues, and cylindrospermopsin. The test uses antibodies that recognise a broad range of analogues for each toxin, potentially making the test more sensitive than LC in some instances where the sample contains very low levels of multiple different congeners in the same family. Multiplexing did not seem to significantly alter each individual test’s sensitivity and toxin can be detected in both algal and seawater samples in 15 min; further testing is on-going to extend this to shellfish samples.

Reverté et al. used the same system to detect TTX [157]. This test uses the same competitive-based format and can detect TTX levels as low as 0.4–3.29 μg/g in pufferfish tissue.

Planar waveguide cartridges are promising tools as second generation lateral flow devices for the robust, on site detection of marine biotoxins; they are rapid, sensitive, and multiple toxins can be screened on a single plastic cartridge. The cartridges are single use; they cannot be regenerated, which raises concerns about the amount of plastic waste generated by multiple samples. Furthermore, due to the fluorescent nature of these tests, they require a special reader to analyze the results. That said, both of these concerns can be addressed as the technology matures and miniaturization occurs.

### 5.2. Enzyme Inhibition-Based Biosensors

Campàs and Marty haven taken a different approach, utilizing protein phosphatase 2A (PP2A) immobilized to a polymer-coated graphite electrode to detect OA in algal samples. PP2A is the biological target of OA; the reversible inhibition of PP2A is the mechanism of action of OA. The authors were able to measure PP2A activity using chronoamperometry as an indirect measurement of OA levels [158]. Similarly, Zhou et al. developed another alternative approach for the detection of OA using PP2A inhibition [159]. Their approach utilized disposable carbon nanotubes on a screen-printed electrode containing immobilized PP2A for assessing mussels. The carbon nanotube method offered a greater dynamic range of 1300 µg/L, with an LOD of 0.55 µg/L.

### 5.3. Aptamers-Based Biosensor 

Aptamer-based biosensors (aptasensors), are emerging as one of the most high-throughput, sensitive and specific POST methods, and most recently it has been pointed out as one of the best candidates for marine toxin detection [160]. Therefore, aptamers are a potential synthetic solution to replace receptors and antibodies and whereby electrochemical techniques have been shown to be more successful in their application. Gao et al. combined a biolayer inferometry (BLI) with aptamers to detect STX, GTXs, and PLTX [161,162,163]. These again are competitive-based assays, whereby toxin is immobilised onto the biosensor surface and signal is inversely proportional to the amount of toxin in the sample. Uniquely, Gao, et al. used HRP-labelled aptamers as biorecognition reporters. Aptamers are smaller than monoclonal antibodies (mAbs) and thus may be used to increase signal efficiency [161]. Most recently, Chinnappan et al. reported robust and highly sensitive graphene oxide (GO) as the fluorescence sensing platform for probing the high affinity of an aptamer for the detection of CYN toxin from water. In this study, it has been shown that the limit of detection using a short derived aptamer (obtained from a long wild-type CYN-sensing aptamer) is 6-fold lower (17 pM) than the longer aptamer [164]. The current development of aptasensors has reached the stage of ultra-sensitivity and amongst the others are one of the most prospective biosensor systems [160]. For example, Qiange et al. demonstrated an ultra-sensitive (LOD 3 fg/mL) and fast (30 min) colorimetric gold nanoparticle aptasensor for the detection of STX from water [165].

## 6. Prospective Trends and Technologies 

There are a number of emergent technologies that can also be applied to marine toxin detection and some of these are adaptations to current methods. Among the other POST methods, detection using a smartphone has been reviewed most recently for food safety applications as a sensitive prospective technology as the smartphone can be used as a portable, handheld reader for field tests. Additional filters or lenses can be added to the camera to improve image quality, and software or apps can be designed to interpret data, display results, and backup information to the cloud [166]. Moreover, two other studies reported the development of promising smartphones technologies to detect OA and STX biotoxins with two different homemade applications iStrip [167] and iPlate [168]. In the study performed by Fang et al. a smartphone-based system connecting with competitive immunoassay strips was designed as a robust POST method to detect these two biotoxins with high sensitivity and specificity (evaluated LOD was 2.8 ng/mL for OA and 9.8 ng/mL for STX) [167]. In turn, the approach shown by Su et al. reported an improved and sensitive field applicable biochemical detection method, as an indirect competitive ELISA assay, with a smartphone based portable system as a Bionic e-Eye [168].

Other groups are miniaturizing current technology to make it more portable and accessible on-site. Traditional SPR equipment, as well as analytical detection instruments and ELISA plate readers are all bulky, heavy, and impossible to use in the field. Chinowsky et al. have miniaturized the technology and developed a SPR the size of a small briefcase that can be used for field research [169]. Most recently, miniaturized micro HPLC (µHPLC) or LC systems were developed to perform high-throughput analysis at point-of-care. These new systems would have indisputable advantages for use in marine toxin detection due to their shorter time of analysis (increased separation speed) and lower costs (low samples or reagents consumption) of analysis than traditional HPLC and LC systems [170]. Additionally, the traditional ELISA plate reader has also a POST replacement. Jensen et al. developed a miniaturized ELISA plate reader for measuring the optical density of 96-well plates. The advantages of this equipment are its small size, sensitivity and due to the addition of a wireless communication module there is the possibility to monitor multiple devices in real-time [171]. Another interesting approach which might replace the traditional ELISA plate reader for marine toxin detection was reported by Berg et al. who developed a handheld, cost-effective, smartphone-based colorimetric microplate reader. This instrument uses a 3D-printed opto-mechanical attachment to hold and illuminate a 96-well plate using a light-emitting-diode (LED) array. Light is transmitted through each well, and is then collected via 96 individual optical fibers [172]. The benefits of this user-friendly microplate reader is the speed of analysis (1 min), accuracy and interpretation obtained results using a smartphone [172].

Some groups are looking at completely different diagnostic approaches, which rely on selection and application of different kinds of biorecognition molecules. For example, instead of using antibodies, phage-derived peptides have been explored for marine toxin detection in the past [173,174,175]. Since the first description of phage-display was in 1985, this technology evolved and revolutionized drug discovery, biomolecular interaction, enzymes optimization and development of biosensors through the development of biorecognition molecules [176]. This technology enables us to discover completely new molecules, such as peptides, other binding proteins or scaffolds, which can be used for a selected target and applied on sensor platform with high efficacy [176]. An interesting approach was reported by Shriver-Lake et al. for the detection of two marine biotoxins (DA and STX) where a recombinant antibody (scFv) was applied for the sensitive and specific detection within an xMAP assay (microsphere-based competitive immunoassay) [177]. Another promising approach using recombinant antibodies and a Lab-On-A-Disc (LOAD) platform was reported by Maguire et al. for the detection of DA and STX. This platform was reported to detect both marine biotoxins with high sensitivity and specificity with an analysis time of 30 min [178]. Many groups are currently investigating other biorecognition molecules such as aptamers, for use in marine toxin detection [179,180,181,182], whereby the selection of these biomolecules is obtained through the Systematic evolution of ligands by exponential enrichment (SELEX) process. It is possible to replace antibodies in traditional immunoassay formats with these types of small molecules, or to use them to develop new types of assays, such as the open-sandwich immunoassay for PSP toxin detection [175].

Another prospective new trend in the detection of marine biotoxins is the use of synthesized, novel nanoparticles to develop nanosensors and other assays. The use of nanomaterials means that electrochemical biosensors can be miniaturized and become portable, an important criterion for on-site screening. Although there is still uncertainty regarding the best strategies for assay development e.g., which nanomaterials to use or how to best immobilize antibodies, electrochemical biosensors present a promising candidate for rapid on-site screening for marine toxins in shellfish. Specific properties including their compact nature, relatively quick assay time, capacity for regeneration, and high sensitivity make them ideal for on-site use. Nelis et al. has shown a proof-of-concept for the detection of domoic acid evaluating different nanomaterials for this purpose using the Psalmsens portable sensor [183] and furthermore that it can be applied for the detection of okadaic acid [184]. Though futuristically where this type of sensor might excel would be in the environmental sample processors for remote sensing. Another study performed by Gholami et al. [185] pointed out the development of a nanosensor for the sensitive and specific detection of maitotoxin (MTX) using novel synthesized carbon quantum dots (CQDs) and gold nanoparticles (AuNPs) using fluorescence resonance energy transfer (FRET) as a sensing method. The main advantages of this sensor was the high specificity for MTX detection from matrix and a LOD, which was evaluated to be 1−600 pmol/L and 0.3 pmol/L [185]. 

There have been efforts to circumvent the requirement of toxin standard as an immunogen; for example, through the generation of anti-idiotypic antibodies. Anti-idiotypic antibodies are raised against antibodies that are specific to the analyte of interest. As such, anti-idiotypic antibodies may present chemical structures similar to the analyte, perhaps providing a suitable substitute for competitive assays [186,187,188]. Advantages of the use anti-idiotypic antibodies have been recently pointed out by Schulz et al. where these biorecognition molecules were used for an electrochemical multiplex (fiveplex) biochip assay for the detection of low molecular weight toxins including STX with high sensitivity (1.2 ng/mL) and recovery [189].

## 7. Challenges for Sample Preparation

Noticeably, there is a lack of emergent multiplex technology. Shellfish can be contaminated with multiple toxins at the same time and multi-toxin detection in a single assay such as an electronic mouse would be hugely beneficial to shellfish producers and consumers [190]. Though some limitations in the evaluation of new biosensor approaches for this application can be in the accessibility to suitable antibodies or alternative binders and sufficient toxin for the preparation of competitive chip surfaces through direct conjugation of the toxin or the use of toxin protein conjugates. 

In addition the shellfish matrix and chemistry of marine toxins is complex, and thus, it can be difficult to isolate particular analytes of interest. Non-specific moieties can inhibit or mask target analytes, making them difficult to detect, or conversely enhance the toxin signal, over-estimating toxin presence. As such, toxin extraction in the sample preparation is a crucial first step in any detection assay to ensure the target analyte is available for recognition. This is further complicated in multiplex assays; shellfish toxins exhibit key differences in their solubility: OA, DTXs, and AZAs are lipophilic, whereas, PSTs, and DA are hydrophilic. Furthermore, different toxin derivatives within each group can exhibit varied solubility [191]. Solvent-based extractions are widely used, where shellfish tissue is homogenised and extracted with a solvent. Importantly, for multiplex assays, the choice of solvent must ensure that it isolates each of these toxin groups and all the relevant analogues.

Alcohol-based solvent extraction is one of the most common methods when extracting toxins from shellfish flesh prior to analysis. Methanol has been used widely for extraction of regulated lipophilic toxins such as OA, DTXs, AZAs, YTXs and PTXs, as well as other lipophilic toxin groups such as the cyclic imines and palytoxins. Detection of OA-group esters requires an additional alkaline hydrolysis step to account for the added acetyl group [101,117,128]. Alternatively, ethanol has been used to extract OA, DA, and STX for detection by LC-MS/MS and ELISA [81]. Jawaid et al. were able to extract DA and STX using distilled water for detection by LFIA [125,127]. In terms of extraction protocols that are practical for portable, field-based applications, distilled water or simple buffer solutions would be ideal extraction mediums, whereas alcohol-based solvents of appropriate purity are highly flammable controlled substances and cannot be easily acquired by shellfish growers or novice end users and are more difficult for distribution in kits.

Studies assessing multiple toxins in shellfish often utilize SPE for both hydrophilic toxin [59,192] and lipophilic toxin extraction [193]. These are not practical however for on-site applications.

Traditional SPE can prove ineffective when the analyte and other matrix components share chemical or physical characteristics, or when they are extremely contaminated. Immunoaffinity columns (IACs) use monoclonal antibodies (mAbs) to enrich analytes, potentially resulting in a more sensitive analysis. Due to the highly specific nature of mAbs, IACs can remove the majority of interfering matrix elements whilst also enhancing the availability of the toxins. Puech et al. developed an IAC for the extraction of DSP toxins in shellfish, prior to analysis by high performance LC-fluorimetry [194]. Similarly, Chen et al. have made an IAC for enriching DA prior to LC-MS/MS analysis [195]. In a slightly different approach, Devlin et al. coupled an anti-STX antibody to hollow glass magnetic microspheres in order to enrich mussel samples for detection by high performance LC (HPLC) [196]. A similar approach has also been used for determining STX levels in human urine [197]. IAC technology is very promising, however, mAbs are expensive to develop and produce, which limits the IAC’s potential. Additionally, SPE in general still requires specialised equipment and can be labour intensive. 

After SPE, many protocols incorporate a dilution step prior to the assay. This ensures that the toxin concentration is within the assay’s dynamic range whilst also further diluting non-specific molecules to reduce matrix interference. However, this leads to another significant hurdle to the development of multiplex assays: the disparity between the action levels that have been set for each toxin. For example, OA-group toxins have an action level of 160 µg/kg, and DA has an action level of 20,000 µg/kg; it can be difficult to ascertain a single dilution factor which will simultaneously ensure each toxin is detected with sufficient sensitivity. Therefore, the sample preparation is continuously a bottle neck for the multiplexing of any targets but is more complex in foodstuffs rather than liquids and when varying action levels are set compared to being banned substances like veterinary drug residues in other fields of application. Achieving a simple novice end user preparatory method is key for any of the technologies described but particularly for on site applications where equipment and experience can be limited. 

## 8. Procedural Practicalities for End User Needs

HABs remain a frequent occurrence throughout the world, contaminating shellfish produce, putting consumer health at risk, forcing harvesting site closures, and inflicting substantial economic damage. Currently approved regulatory methods for detection of Lipophilic, PSP and ASP toxins include chemical detection methods such as LC-MS/MS, LC-FLD and LC-UV. While the chromatography-based reference methods are accurate, they are costly, time-consuming, and require specialized equipment with trained personnel. Although the replacement of the mouse bioassay has been achieved which drove this innovation there is a great need for screening methods that follow the QuEChERS principle in being quick, easy, cheap, rugged, and safe to facilitate on-site testing by shellfish producers, to allow them to make early and informed decisions about their produce. Similarly, alternative methods have focused on one toxin family in general and whereby the use of multiple tests on site to cover all toxins is also not practically feasible for the shellfish industry. Ideally, the industry wish to have at their disposal one simple test that covers all toxins and ensures that there product is safe for harvest and distribution. Hence, the drive towards multiplex analysis in this field, but this comes also with additional challenges. 

Presently, commercially available screening methods predominantly use ELISA and LFIA technologies (Table 2). Whilst ELISA offers accuracy and relatively high throughput; it often requires a few hours to complete and it is not particularly feasible as an on-site screening method, given the need to use a range of laboratory equipment, reagents and consumables. Additionally, screening for multiple toxins requires multiple tests to be run, which increases the cost, time, reagents, and amount of sample needed to screen stock. Alternatively, the LFIA is very simple to use, requires only minimal training, and is relatively very quick to perform, in most cases less than 30 min from receiving a sample to obtaining a result. But unfortunately, these tests only offer qualitative or at best semi-quantitative results, and similar to the ELISA, each toxin requires its own device and accompanying sample preparation procedure. New biosensors are being developed and validated to offer rapid, sensitive, and often portable methods of screening shellfish toxins. These include electrochemical sensors, surface plasmon resonance, fluorometry, and microfluidic devices. A summary of these methods is given in Table 3. A majority of these alternatives use highly specific antibody-based recognition of toxins. Immune recognition is not without its limitations; for example, it is very difficult to obtain an antibody that is cross-reactive with all associated toxin analogues to the degree that mirrors their relative toxicity as determined from a mouse bioassay or upcoming oral toxicity studies. Additionally, all toxins do not appear in all regions worldwide (Table 1) and whereby the end user may request custom designed devices for their needs with the ability to detect only a selection of problematic regulated toxins. Furthermore, unfortunately, many of these assays still require expensive, specialized, equipment and are not suitable for rapid, on-site testing.

Most of the proposed screening methods are reliant on the availability of a toxin standard. The standard is often used both as an immunogen for antibody production or directly in competitive inhibition-based assays. This represents a significant hurdle in their development and especially in their production at a commercial scale as certain shellfish toxins are recognized as highly poisonous and thus are highly controlled substances.

Sample preparation is a critical first step to screening shellfish toxins and can be the crucial bottleneck in the development of any method. Sample preparation methods must be relatively simple, quick, and safe if they are to be employed on-site. This greatly limits the options available to test-kit developers when determining which solvents or equipment to use in order to overcome the interferences associated with these complex sample matrices. For example, the current accepted extraction methods for DSP toxins rely on methanol and/or ethanol. However, these are highly flammable controlled substances that are difficult to store and transport. As such, many commercially available kits do not supply extraction solvents, leaving it up to the end user to source and supply their own. This makes testing more onerous, more expensive, and more confusing for shellfish producers. As such, shellfish producers and assay manufacturers alike would benefit from the development of alternative extraction methods. Ideally, new extraction methods would have as few steps as possible, will be easily achievable on site, and will use non-solvent extractions that can be bundled with the assay. This means the methods must be safe to perform without specialist equipment, the solvents must be safe to store in small quantities, and everything must be easily transported—all whilst maintaining great efficiency to extract a majority of the toxins present. Other studies have reported methods to extract multiple groups of toxin from one sample for analysis but whereby different dilutions may need to be applied to the different methods for the different families of toxins to achieve the correct sensitivity [81,195]. 

## 9. Conclusions and Future Outlook

Progress in the field of portable toxin testing for industry is rapid where toxins are available as standards and many promising technologies have been developed, each with their own strengths and weaknesses. Many of these assays would benefit from miniaturization to make them portable and accessible on-site. Furthermore, many of these assays require further testing in new matrices e.g., the full range of bivalve species, gastropods and crustaceans, optimization and validation of protocols to an appropriate standard, and comparison against the current reference methods. It will be important to perform collaborative validation studies that assess interlaboratory performance characteristics and also verify the test kit components are robust and viable across a full range of environments and following transportation. Different shellfish species thrive in different waters across the globe, from freezing cold brackish lakes and estuaries to highly saline, warm tropical lagoons, and testing equipment needs to be able to function under a wide range of environmental conditions to accommodate the different farmed species. For example, environmental temperature, humidity, salinity, and light density are all important conditions that can influence test results. Moreover, saltwater is extremely corrosive, and testing equipment should ideally be water- and corrosion-resistant. It will additionally be vital to consult and validate the tests to the end user requirements for their ability to be fit for purpose on site.

Going forward, it will be most important to design highly accurate quantitative solutions that can give shellfish producers an accurate assessment of contamination levels in shellfish produce on their site at the time of testing. This would allow producers to follow immediate trends and make an informed decision about the state of a current contamination event or even predict an upcoming event. Together, tools designed for practical usage on site will save shellfish producers time and money, protect consumers, boost consumer confidence, and help ensure the availability of freshly caught, toxin-free shellfish. However, these diagnostic tools for toxins should not be considered in isolation: a holistic approach combining satellite imagery, POST rapid diagnostics and other developing tools [24] for HAB detection could aid the understanding of our oceans in relation to climate and environmental conditions moving towards early warning systems that allow enhanced management and control to complement the data generated by official control testing programmes. Suitable infrastructure and handling capacity will also be required on-site to allow these management systems to benefit shellfish farmers. 

## Figures and Tables

**Table 1:** Major shellfish poisoning syndromes showing causative toxins, vectors, and associated health risks to humans.

Poisoning Syndrome	Toxin	Major Toxin Producing Species	No. of Analogues	Vector (MRL a µg/kg)	Key Areas of Occurrence	Short Term Health Consequences	Long Term Health Consequences	References
Amnesic Shellfish Poisoning (ASP)	Domoic Acid (DA)	*Pseudo-nitzschia* spp.	~10	Shellfish (20,000)	United Kingdom, Europe, USA, Mexico, Australia, New Zealand, Canada	Vomiting, diarrhoea, liver inflammation, abdominal pain, confusion, disorientation, memory loss	Anterograde memory deficit, seizures leading to coma and death	[6]
Diarrhetic Shellfish Poisoning (DSP)	Okadaic Acid (OA) & Dinophysistoxins (DTX)	*Dinophysis* spp. *Prorocentrum lima*	~8	Shellfish (160 b)	Worldwide (United Kingdom, Europe, Scandinavia, North & South America, Asia, Australia & New Zealand)	Nausea, vomiting, diarrhoea, abdominal pain accompanied by chills, headache, fever	Gastrointestinal tumour promoter in laboratory animals	[6]
	Azaspiracid (AZA)	*Azadinium* spp.	~60	Shellfish (160)	Ireland, Mediterranean, South America	Diarrhoea, neurotoxic effects	Unknown	[7]
	Yessotoxin (YTX)	*Protoceratium reticulatum* *Lingulodinium polyedra*	~36	Shellfish (3750)	China, Japan	Unknown	Unknown	
	Pectenotoxin (PTX)	*Dinophysis fortii*	~13	Shellfish (160 b)	China, Japan			
Paralytic Shellfish Poisoning (PSP)	Saxitoxin (STX) & Gonyautotoxin (GTX)	*Alexandrium* spp., *Gymnodinium catenatum*, *Pyrodinium* spp.	>57	Shellfish Crustaceans (800 c)	Worldwide (United Kingdom, Europe, Scandinavia, North & South America, Asia, Africa, Australia & New Zealand)	Paraesthesia, drowsiness, incoherent speech, respiratory paralysis leading to death	Unknown	[6]
	Tetrodotoxin (TTX) *	*Marine bacteria* spp.	>10	Gastropods Fish	China, Japan, United Kingdom, Gulf of Mexico, Mediterranean			[8]
Neurotoxic Shellfish Poisoning	Brevetoxin (BTX/Pbtx)	*Karenia* spp.	>12	Shellfish (800)	Florida, Gulf of Mexico, New Zealand	Act on site 5 of the sodium channel receptor. Nausea, diarrhoea, vomiting, numbness of lips, tongue, &throat, muscular aches, fever, chills, abdominal cramping, reduced heart rate, pupil dilation	Unknown	[9,10]
Other	Palytoxin (PLTX) & Ostreocin (OSTD)	*Ostreopsis* spp.	2 (for PLTX)	Fish Crustacean Shellfish (30 d)	Mediterranean (Italy, Spain)	In vitro binds to the Sodium Potassium ATPase Vomiting, diarrhoea, respiratory distress, death	Unknown	[11,12]
	Mascarenotoxin (McTX) *		2					[13]
	Ovatoxins (OVTX) *		9					[13,14,15]
	Gymnodimine *	*Gymnodinium* spp. *Karenia* spp. *Alexandrium ostenfeldii* *Vulcanodinium rugosum*	5	Shellfish	Scandinavia, United Kingdom, Mediterranean	Not fully known. Similar effects to DSP toxins in mice. Interact with nicotinic acetylcholine receptors Effects in humans have not been reported.	Unknown	[7,16,17]
	Spirolides *		16					
	Pinnatoxins (PnTX) *		8					
	Pteriatoxins (PtTX) *		3					
	Prorocentrolides *	*Prorocentrum* spp.	6					
	Spiroprorocentrimines *		TBD					
Ciguatera Fish Poisoning	Ciguatoxin (CTX) *	*Gambierdiscus* spp. *Amphidinum* spp.	~23	Reef Fish Shellfish Giant claims	Caribbean, Indian and Pacific waters in tropical zone, Spain, Portugal	Act on site 5 of the sodium channel receptor. Nausea, vomiting, diarrhoea, paraesthesia, temperature dysesthesia, pain, weakness, bradycardia, hypotension	Recurrent symptoms from months to years of chronic effects	[7,18,19]
	Maitotoxin (MTX)	*Gambierdiscus* spp. *Fukuyoa* spp.	4	Reef fish	Pacific Ocean	Mode of action not fully elucidated. Toxin believed to play a role in CFP.		[14,20,21]

a. As established by the European Commission Regulations No 853/2004; b. Total toxin limit combined OA + DTX + PTX. c. Total toxin limit combined STX and GTX; d. Total toxin limit combined PTX and OSTD; * Toxins that are emerging and/or unregulated at this time.

**Table 2:** The list of available end-product testing technologies.

Method	Additional Materials Required (Not Included in Main Kit)	Time per Sample (Sample Preparation)	Cost ^1^	Complexity ^2^	Pathway to Commercialization
ELISA/RBA	Microtiter plate reader Orbital shaker Clean running water Pipette(s) w. disposable tips Absorbent towels Timer Sample extraction kit ^3^	90 min (15–60 min ^4^)	££	2	Commercially available (Table 1)
LFD	LFD cassette reader Timer Sample extraction kit ^3^	35–45 min (15–60 min ^4^)	£	1	Commercially available (Table 1)
Flow-through immunoassay	Chemiluminescence imaging Flow-based microarray analysis platform (MCR3) Pipette(s) w. disposable tips Sample extraction kit ^3^	20 min (20 min)	££	2	Assays must be validated in single- and multi- laboratory studies MCR3 technology is not portable for on-site testing by end-users
SPR	Biacore™ Q optical biosensor Pipette(s) w. disposable tips Sample extraction kit ^3^	10 min (60 min)	£££	3	Assays have been validated in single-laboratory studies to AOAC standards SPR technology is suitable for use by novice end-users High cost Wizard driven software for use/maintenance
Electrochemical	Electrochemical analyser Pipette(s) w. disposable tips Sample extraction kit ^3^	10–45 min (30–60 min)	££	2	Assays must be validated in single- and multi- laboratory studies Challenging set-up for end users
Planar waveguide	Waveguide reader Pipette(s) w. disposable tips Sample extraction kit ^3^	20 min (60 min)	£	1	Assays must be validated in single- and multi- laboratory studies Software for data analysis not suitable for novice end user

^1^ £ = Minimal additional equipment required, ££ = Some specialised equipment required, £££ = Expensive, specialised equipment required; ^2^ 1 = No training or facilities required, 2 = Minimal training and facilities required, 3 = Technical staff and facilities required; ^3^ Common requirements include a blender, weighing scales, a roller mixer, a centrifuge, a hot plate or hot bath, filters. Extraction buffers vary dependent on the assay (i.e., varying concentrations of NaAc, MeOH, Acetic Acid, or NaOH with HCl); ^4^ Many DSP kits require a 60-min alkaline hydrolysis step to accurately assess DSP concentration.

**Table 3:** A list of commercially available marine toxin detection kits.

			Toxin							
Company	Product	Type	DA	OA	DTXs	STX	GTXs	TTX	BTXs	CTXs
Bioo Scientific	MaxSignal^®®^ Domoic Acid	ELISA	√ ☐							
	MaxSignal^®®^ Okadaic Acid	ELISA		√ ☐	*					
	MaxSignal^®®^ Saxitoxin	ELISA				√ ☐	*			
Zeulab	DomoTest	ELISA								
	OkaTest	RBA		√ ☐	*					
	Saxitest	ELISA								
Creative Diagnostics	Domoic Acid Kit	ELISA	√ ☐							
	Tetrodotoxin Kit	ELISA						√ ☐		
Mercury Science	Domoic Acid Kit	ELISA	√ ☐							
	Domoic Acid Field Kit	DOT ^1^	√ ☐							
	Total Saxitoxin Kit	ELISA				√ ☐	*			
Abraxis	Domoic Acid ELISA Kit	ELISA	√ ☐							
	Okadaic acid ELISA Kit	ELISA		√ ☐	*					
	Okadaic Acid PP2A Kit	RBA		√ ☐	√ ☐					
	Saxitoxins Shipboard Kit	ELISA				√ ☐	−			
	Brevetoxin (NSP) Test	ELISA							√ ☐	
Marbionc	Brevetoxin ELISA Kit	ELISA							√ ☐	
	Brevetoxin/Ciguatoxin Kit	RBA							√ ☐	√ ☐
Unibiotest	Tetrodotoxin ELISA Test	ELISA						√ ☐		
	Tetrodotoxin Rapid Test	LFIA						√ ☐		
Beacon	Saxitoxin ELISA kit	ELISA				√ ☐	√ ☐			
R-Biopharm	EuroProxima Domoic Acid	ELISA	√ ☐							
	EuroProxima Okadaic Acid	ELISA		√ ☐	√ ^2^					
	EuroProxima Saxitoxin	ELISA				√ ☐	√ ☐			
	EuroProxima Tetrodotoxin	ELISA						√ ☐		
Biosense^®®^ Laboratories	ASP ELISA Kit	ELISA	√ ☐							
	DSP ELISA kit	ELISA		√ ☐	−					
	PSP ELISA kit	ELISA				√ ☐	−			
Neogen	Reveal^®®^ 2.0 for ASP	LFIA	√ ☐							
	Reveal^®®^ 2.0 for DSP	LFIA		√ ☐	√ ☐					
	Reveal^®®^ 2.0 for PSP	LFIA				√ ☐	*			
Scotia	ASP Test	LFIA	√ ☐							
	DSP Test	LFIA		√ ☐	*					
	PSP Test	LFIA				√ ☐	*			

^1^ A dot blot assay functions similar to an ELISA but on a membrane rather than a well. ^2^ DTX-1 and DTX-2, but not DTX-3 √ Kit available for detecting toxins − Not able to detect toxins * Information unavailable.

## Data Availability

Not applicable.

## References

[1] Food and Agriculture Organization of the United Nations (2020). Sustainability in action. The State of World Fishery and Aquaculture 2020.

[2] Simoes A.J.G., Haldogo C.A. (2017). The Economic Complexity Observatory: An Analytical Tool for Understanding the Dynamics of Economic Development. https://oec.world/en/profile/hs92/0307/#Exporters.

[3] Food and Agriculture Organization of the United Nations GLOBEFISH—Information and Analysis on World Fish Trade. http://www.fao.org/in-action/globefish/market-reports/resource-detail/en/c/1176312/.

[4] CDC. https://www.cdc.gov/habs/general.html.

[5] Ralston E.P., Kite-Powell H., Beet A. (2011). An estimate of the cost of acute health effects from food- and water-borne marine pathogens and toxins in the USA. J. Water Health.

[6] Anderson D. HABs in a changing world: A perspective on harmful algal blooms, their impacts, and research and management in a dynamic era of climatic and environmental change. Proceedings of the 15th International Conference on Harmful Algae, Changwon.

[7] Botana L.M. (2008). Seafood and Freshwater Toxins: Pharmacology, Physiology, and Detection.

[8] Turner A.D., Dhanji-Rapkova M., Coates L., Bickerstaff L., Milligan S., O’Neill A., Faulkner D., McEneny H., Baker-Austin C., Lees D.N. (2017). Detection of Tetrodotoxin Shellfish Poisoning (TSP) Toxins and Causative Factors in Bivalve Molluscs from the UK. Mar. Drugs.

[9] Davidson K., Baker C., Higgins C., Higman W., Swan S., Veszelovszki A., Turner A.D. (2015). Potential Threats Posed by New or Emerging Marine Biotoxins in UK Waters and Examination of Detection Methodologies Used for Their Control: Cyclic Imines. Mar. Drugs.

[10] Reynolds D.A., Yoo M.-J., Dixson D.L., Ross C. (2020). Exposure to the Florida red tide dinoflagellate, Karenia brevis, and its associated brevetoxins induces ecophysiological and proteomic alterations in Porites astreoides. PLoS ONE.

[11] Ciminiello P., Dell’Aversano C., Fattorusso E., Forino M. (2010). Palytoxins: A still haunting Hawaiian curse. Phytochem. Rev..

[12] Ciminiello P., Dell’Aversano C., Iacovo E.D., Fattorusso E., Forino M., Tartaglione L. (2011). LC-MS of palytoxin and its analogues: State of the art and future perspectives. Toxicon.

[13] Ramos V., Vasconcelos V. (2010). Palytoxin and Analogs: Biological and Ecological Effects. Mar. Drugs.

[14] Ajani P., Harwood D.T., Murray S.A. (2017). Recent Trends in Marine Phycotoxins from Australian Coastal Waters. Mar. Drugs.

[15] Tartaglione L., Iacovo E.D., Mazzeo A., Casabianca S., Ciminiello P., Penna A., Dell’Aversano C. (2017). Variability in Toxin Profiles of the Mediterranean Ostreopsis cf. ovata and in Structural Features of the Produced Ovatoxins. Environ. Sci. Technol..

[16] Amar M., Aráoz R., Iorga B.I., Yasumoto T., Servent D., Molgó J. (2018). Prorocentrolide-A from Cultured Prorocentrum lima Dinoflagellates Collected in Japan Blocks Sub-Types of Nicotinic Acetylcholine Receptors. Toxins.

[17] Moreiras G., Leão J.M., Gago-Martínez A. (2019). Analysis of Cyclic Imines in Mussels (*Mytilus galloprovincialis*) from Galicia (NW Spain) by LC-MS/MS. Int. J. Environ. Res. Public Health.

[18] Díaz-Asencio L., Clausing R.J., Vandersea M., Chamero-Lago D., Gómez-Batista M., Hernández-Albernas J.I., Chomérat N., Rojas-Abrahantes G., Litaker R.W., Tester P. (2019). Ciguatoxin Occurrence in Food-Web Components of a Cuban Coral Reef Ecosystem: Risk-Assessment Implications. Toxins.

[19] Farrell H., Murray S.A., Zammit A., Edwards A.W. (2017). Management of Ciguatoxin Risk in Eastern Australia. Toxins.

[20] Boente-Juncal A., Álvarez M., Antelo Á., Rodríguez I., Calabro K., Vale C., Thomas O.P., Botana L.M. (2019). Structure Elucidation and Biological Evaluation of Maitotoxin-3, a Homologue of Gambierone, from *Gambierdiscus belizeanus*. Toxins.

[21] Pisapia F., Holland W.C., Hardison D.R., Litaker R.W., Fraga S., Nishimura T., Adachi M., Nguyen-Ngoc L., Séchet V., Amzil Z. (2017). Toxicity screening of 13 Gambierdiscus strains using neuro-2a and erythrocyte lysis bioassays. Harmful Algae.

[22] Doucette G.J., Medlin L.K., McCarron P., Hess P. (2018). Detection and Surveillance of Harmful Algal Bloom Species and Toxins. Harmful Algal Blooms.

[23] Medlin L.K., Orozco J. (2017). Molecular Techniques for the Detection of Organisms in Aquatic Environments, with Emphasis on Harmful Algal Bloom Species. Sensors.

[24] Medlin L., Gamella M., Mengs G., Serafín V., Campuzano S., Pingarrón J.M. (2020). Advances in the Detection of Toxic Algae Using Electrochemical Biosensors. Biosensors.

[25] Blondeau-Patissier D., Gower J.F., Dekker A.G., Phinn S.R., Brando V.E. (2014). A review of ocean color remote sensing methods and statistical techniques for the detection, mapping and analysis of phytoplankton blooms in coastal and open oceans. Prog. Oceanogr..

[26] Malthus T.J., Lehmann E., Ho X., Botha E., Anstee J. (2019). Implementation of a Satellite Based Inland Water Algal Bloom Alerting System Using Analysis Ready Data. Remote. Sens..

[27] Malthus T.J., Ohmsen R., Van Der Woerd H.J. (2020). An Evaluation of Citizen Science Smartphone Apps for Inland Water Quality Assessment. Remote. Sens..

[28] European Commission (2004). Regulation (EC) No 853/2004 of the European Parlamient and of the Council of 29 April 2004 Laying down Specific Hygiene Rules for on the Hygiene of Foodstuffs.

[29] European Commission (2005). Regulation (EC) No 2074/2005: Laying down Implementing Measures for Certain Products under Regulation (EC) No 853/2004 of the European Parliament and of the Council and for the Organisation of Official Controls under Regulation (EC) No 854/2004 of the European Parliament and of the Council and Regulation (EC) No 882/2004 of the European Parliament and of the Council, Derogating from Regulation (EC) No 852/2004 of the European Parliament and of the Council and Amending Regulations (EC) No 853/2004 and (EC) No 854/2004.

[30] European Commission (2006). Regulation (EC) No 1664/2006: Amending Regulation 2074/2005 with Regards to Measures for Certain Products, in Particular the Testing Method for Paralytic Shellfish Poison (PSP).

[31] European Commission (2011). Commission Regulation (EU) No 15/2011 of 10 January 2011 amending Regulation (EC) No 2074/2005 as Regards Recognised Testing Methods for Detecting Marine Biotoxins in Live Bivalve Molluscs.

[32] European Commission (2017). Commission Regulation (EU) 2017/1980 of 31 October 2017 amending Annex III to Regulation (EC) No 2074/2005 as Regards Paralytic Shellfish Poison (PSP) Detection Method.

[33] Food and Agriculture Organization of the United Nations and World Health Organization Toxicity Equivalency Factors for Marine Biotoxins Associated with Bivalve Molluscs. https://apps.who.int/iris/bitstream/handle/10665/250663/9789241511483-eng.pdf;jsessionid=8C3485B7D71998D8D0E870446619B97D?sequence=1.

[34] Grasl-Kraupp B., Hogstrand C., Hoogenboom L., Nebbia C.S., Oswald I.P., Rose M., Roudot A.-C., Schwerdtle T., Vleminckx C., Vollmer G. (2017). Scientific opinion on the risks for public health related to the presence of tetrodotoxin (TTX) and TTX analogues in marine bivalves and gastropods. EFSA J..

[35] Gobler C.J., Koch F., Kang Y., Berry D.L., Tang Y.Z., Lasi M., Walters L., Hall L., Miller J.D. (2013). Expansion of harmful brown tides caused by the pelagophyte, Aureoumbra lagunensis DeYoe et Stockwell, to the US east coast. Harmful Algae.

[36] Koch F., Kang Y., Villareal T.A., Anderson D.M., Gobler C.J. (2014). A Novel Immunofluorescence Flow Cytometry Technique Detects the Expansion of Brown Tides Caused by Aureoumbra lagunensis to the Caribbean Sea. Appl. Environ. Microbiol..

[37] Kudela R.M., Gobler C.J. (2012). Harmful dinoflagellate blooms caused by *Cochlodinium* sp.: Global expansion and ecological strategies facilitating bloom formation. Harmful Algae.

[38] McCarthy M., Bane V., García-Altares M., van Pelt F.N., Furey A., O’Halloran J. (2015). Assessment of emerging biotoxins (pinnatoxin G and spirolides) at Europe’s first marine reserve: Lough Hyne. Toxicon.

[39] Rhodes L. (2011). World-wide occurrence of the toxic dinoflagellate genus Ostreopsis Schmidt. Toxicon.

[40] Zhang Q.-C., Qiu L.-M., Yu R.-C., Kong F.-Z., Wang Y.-F., Yan T., Gobler C.J., Zhou M.-J. (2012). Emergence of brown tides caused by Aureococcus anophagefferens Hargraves et Sieburth in China. Harmful Algae.

[41] Campbell K., Vilariño N., Botana L.M., Elliott C.T. (2011). A European perspective on progress in moving away from the mouse bioassay for marine-toxin analysis. TrAC Trends Anal. Chem..

[42] Wiese M., D’Agostino P.M., Mihali T.K., Moffitt M.C., Neilan B.A. (2010). Neurotoxic Alkaloids: Saxitoxin and Its Analogs. Mar. Drugs.

[43] Ofuji K., Satake M., McMahon T., James K.J., Naoki H., Oshima Y., Yasumoto T. (2001). Structures of Azaspiracid Analogs, Azaspiracid-4 and Azaspiracid-5, Causative Toxins of Azaspiracid Poisoning in Europe. Biosci. Biotechnol. Biochem..

[44] Rehmann N., Hess P., Quilliam M.A. (2008). Discovery of new analogs of the marine biotoxin azaspiracid in blue mussels (*Mytilus edulis*) by ultra-performance liquid chromatography/tandem mass spectrometry. Rapid Commun. Mass Spectrom..

[45] Rossi R., Dell’Aversano C., Krock B., Ciminiello P., Percopo I., Tillmann U., Soprano V., Zingone A. (2017). Mediterranean Azadinium dexteroporum (*Dinophyceae*) produces six novel azaspiracids and azaspiracid-35: A structural study by a multi-platform mass spectrometry approach. Anal. Bioanal. Chem..

[46] Turnbull A.R., Tan J.Y.C., Ugalde S.C., Hallegraeff G.M., Campbell K., Harwood D.T., Dorantes-Aranda J.J. (2018). Single-Laboratory Validation of the Neogen Qualitative Lateral Flow Immunoassay for the Detection of Paralytic Shellfish Toxins in Mussels and Oysters. J. AOAC Int..

[47] European Commission (2014). Amending Regulation (EC) No 401/2006 as Regards Methods of Sampling of Large Lots, Spices and Food Supplements, Performance Criteria for T-2, HT-2 Toxin and Citrinin and Screening Methods of Analysis.

[48] Yasumoto T., Oshima Y., Yamaguchi M. (1978). Occurrence of a new type of shellfish poisoning in the Tohoku district. Nippon Suisan Gakkaishi.

[49] Hollingworth T., Wekell M.M., Hellrich K. (1990). Official Methods of Analysis of the AOAC.

[50] EU Directive (2010). Directive 2010/63/EU of the European Parliament and of the Council on the Protection of Animals Used for Scientific Purposes.

[51] Botana L.M., Vilariño N., Alfonso A., Vale C., Louzao C., Elliott C.T., Campbell K., Botana A.M. (2010). The problem of toxicity equivalent factors in developing alternative methods to animal bioassays for marine-toxin detection. TrAC Trends Anal. Chem..

[52] Council of the European Union (1997). Council Directive 97/61/EC of 20 October 1997 Amending the Annex to Directive 91/492/EEC Laying down the Health Conditions for the Production and Placing on the Market of Live Bivalve Molluscs.

[53] EU-RL-MB EU-Harmonised Standard Operating Procedure for Determination of Domoic Acid in Shellfish and Finfish by RP-HPLC Using UV Detection. 12; 2008. http://aesan.msssi.gob.es/CRLMB/docs/docs/procedimientos/EU-Harmonised-SOP-ASP-HPLC-UV_Version1.pdf.

[54] EU-RL-MB EU-Harmonised Standard Operating Procedure for Determination of Lipophilic Marine Biotoxins in Molluscs by LC-MS/MS. 31; 2015. https://doi.org/www.aesan.msps.es/en/CRLMB/web/home.shtml.

[55] Lawrence J.F., Niedzwiadek B. (2001). Quantitative Determination of Paralytic Shellfish Poisoning Toxins in Shellfish by Using Prechromatographic Oxidation and Liquid Chromatography with Fluorescence Detection. J. AOAC Int..

[56] Lawrence J.F., Niedzwiadek B., Menard C., De Astudillo L.R., Biré R., A Burdaspal P., Ceredi A., Davis B., Dias E., Eaglesham G. (2005). Quantitative Determination of Paralytic Shellfish Poisoning Toxins in Shellfish Using Prechromatographic Oxidation and Liquid Chromatography with Fluorescence Detection: Collaborative Study. J. AOAC Int..

[57] Rourke W.A., Murphy C.J., Pitcher G., Van De Riet J.M., Burns B.G., Thomas K.M., A Quilliam M. (2008). Rapid Postcolumn Methodology for Determination of Paralytic Shellfish Toxins in Shellfish Tissue. J. AOAC Int..

[58] Suzuki T., Uchida H., Watanabe R. (2017). LC/MS Analysis of Marine Toxins. Advances in Ion Mobility-Mass Spectrometry: Fundamentals, Instrumentation and Applications.

[59] Rodríguez I., Alfonso A., González-Jartín J.M., Vieytes M.R., Botana L.M. (2018). A single run UPLC-MS/MS method for detection of all EU-regulated marine toxins. Talanta.

[60] Boundy M.J., Selwood A.I., Harwood D.T., McNabb P.S., Turner A.D. (2015). Development of a sensitive and selective liquid chromatography–mass spectrometry method for high throughput analysis of paralytic shellfish toxins using graphitised carbon solid phase extraction. J. Chromatogr. A.

[61] Quilliam M.A., Xie M., Hardstaff W.R. (1995). Rapid Extraction and Cleanup for Liquid Chromatographic Determination of Domoic Acid in Unsalted Seafood. J. AOAC Int..

[62] These A., Scholz J., Preiss-Weigert A. (2009). Sensitive method for the determination of lipophilic marine biotoxins in extracts of mussels and processed shellfish by high-performance liquid chromatography–tandem mass spectrometry based on enrichment by solid-phase extraction. J. Chromatogr. A.

[63] Gerssen A., McElhinney M.A., Mulder P.P.J., Bire R., Hess P., De Boer J. (2009). Solid phase extraction for removal of matrix effects in lipophilic marine toxin analysis by liquid chromatography-tandem mass spectrometry. Anal. Bioanal. Chem..

[64] Botana L.M., Alfonso A., Botana A., Vieytes M.R., Vale C., Vilariño N., Louzao C. (2009). Functional assays for marine toxins as an alternative, high-throughput-screening solution to animal tests. TrAC Trends Anal. Chem..

[65] Doucette G.J., Logan M.M., Ramsdell J.S., Van Dolah F.M. (1997). Development and preliminary validation of a microtiter plate-based receptor binding assay for paralytic shellfish poisoning toxins. Toxicon.

[66] Van Dolah F.M., A Leighfield T., Doucette G.J., Bean L., Niedzwiadek B., Rawn D.F.K. (2010). Single-laboratory validation of the microplate receptor binding assay for paralytic shellfish toxins in shellfish. J. Assoc. Off. Anal. Chem..

[67] Van Dolah F.M., E Fire S., A Leighfield T., Mikulski C.M., Doucette G.J. (2012). Determination of Paralytic Shellfish Toxins in Shellfish by Receptor Binding Assay: Collaborative Study. J. AOAC Int..

[68] Turner A.D., Broadwater M., Van Dolah F. (2018). Use of the receptor binding assay for determination of paralytic shellfish poisoning toxins in bivalve molluscs from Great Britain and the assessment of method performance in oysters. Toxicon.

[69] Alonso E., Alfonso A., Vieytes M.R., Botana L.M. (2015). Evaluation of toxicity equivalent factors of paralytic shellfish poisoning toxins in seven human sodium channels types by an automated high throughput electrophysiology system. Arch. Toxicol..

[70] Bialojan C., Takai A. (1988). Inhibitory effect of a marine-sponge toxin, okadaic acid, on protein phosphatases. Specificity and kinetics. Biochem. J..

[71] Zeulab (2020). Okatest. https://www.zeulab.com/en/producto/water-and-marine-toxins/enzymatic-water-and-marine-toxins/okatest/.

[72] Smienk H.G.F., Calvo D., Razquin P., Domínguez E., Mata L. (2012). Single Laboratory Validation of A Ready-to-Use Phosphatase Inhibition Assay for Detection of Okadaic Acid Toxins. Toxins.

[73] Smienk H., Domínguez E., Rodríguez-Velasco M.L., Clarke D., Kapp K., Katikou P., Cabado A.G., Otero A., Vieites J.M., Razquin P. (2013). Quantitative Determination of the Okadaic Acid Toxins Group by a Colorimetric Phosphatase Inhibition Assay: Interlaboratory Study. J. AOAC Int..

[74] Aráoz R., Nghiêm H.-O., Rippka R., Palibroda N., De Marsac N.T., Herdman M. (2005). Neurotoxins in axenic oscillatorian cyanobacteria: Coexistence of anatoxin-a and homoanatoxin-a determined by ligand-binding assay and GC/MS. Microbiology.

[75] Aráoz R., Vilariño N., Botana L.M., Molgó J. (2010). Ligand-binding assays for cyanobacterial neurotoxins targeting cholinergic receptors. Anal. Bioanal. Chem..

[76] Fonfría E.S., Vilariño N., Espiña B., Louzao M.C., Alvarez M., Molgó J., Aráoz R., Botana L.M. (2010). Feasibility of gymnodimine and 13-desmethyl C spirolide detection by fluorescence polarization using a receptor-based assay in shellfish matrixes. Anal. Chim. Acta.

[77] Rodríguez L.P., Vilariño N., Molgó J., Araoz R., Botana L.M. (2013). High-throughput receptor-based assay for the detection of spirolides by chemiluminescence. Toxicon.

[78] Hardison D.R., Holland W.C., McCall J.R., Bourdelais A.J., Baden D.G., Darius H.T., Chinain M., Tester P.A., Shea D., Quintana H.A.F. (2016). Fluorescent Receptor Binding Assay for Detecting Ciguatoxins in Fish. PLoS ONE.

[79] Pelin M., Sosa S., Brovedani V., Fusco L., Poli M., Tubaro A. (2018). A Novel Sensitive Cell-Based Immunoenzymatic Assay for Palytoxin Quantitation in Mussels. Toxins.

[80] Garthwaite I. (2000). Keeping shellfish safe to eat: A brief review of shellfish toxins, and methods for their detection. Trends Food Sci. Technol..

[81] Dubois M., Demoulin L., Charlier C., Singh G., Godefroy S., Campbell K., Elliott C., Delahaut P. (2010). Development of ELISAs for detecting domoic acid, okadaic acid, and saxitoxin and their applicability for the detection of marine toxins in samples collected in Belgium. Food Addit. Contam. Part A.

[82] Garthwaite I., Ross K.M., O Miles C., Briggs L.R., Towers N.R., Borrell T., Busby P. (2001). Integrated Enzyme-Linked Immunosorbent Assay Screening System for Amnesic, Neurotoxic, Diarrhetic, and Paralytic Shellfish Poisoning Toxins Found in New Zealand. J. AOAC Int..

[83] McLeod C., Burrell S., Holland P. Review of the Currently Available Field Methods for Detection of Marine Biotoxins in Shellfish Flesh. 86; September 2015. http://www.foodstandards.gov.scot/review-currently-available-field-methods-detection-marine-biotoxins-shellfish-flesh.

[84] Johnson H.M., Frey P.A., Angelotti R., Campbell J.E., Lewis K.H. (1964). Haptenic properties of paralytic shellfish poison conjugated to proteins by formaldehyde treatment. Proc. Soc. Exp. Biol. Med..

[85] Cembella A.D., Parent Y., Jones D., Lamoureux G. Specificity and cross-reactivity of an absorption-inhibition enzyme-linked immunoassay for the detection of paralytic shellfish toxins. Proceedings of the 4th International Conference on Toxic Marine Phytoplankton.

[86] Chu F.S., Fan T.S.L. (1985). Indirect Enzyme-Linked Immunosorbent Assay for Saxitoxin in Shellfish. J. Assoc. Off. Anal. Chem..

[87] Renz V., Terplan G. (1988). En enzymimmunologischer nachweis von saxitoxin. Arch. Fur Lebensm..

[88] Chu F.S., Hsu K.-H., Huang X., Barrett R., Allison C. (1996). Screening of Paralytic Shellfish Posioning Toxins in Naturally Occurring Samples with Three Different Direct Competitive Enzyme-Linked Immunosorbent Assays. J. Agric. Food Chem..

[89] Huang X., Hsu K.-H., Chu F.S. (1996). Direct Competitive Enzyme-Linked Immunosorbent Assay for Saxitoxin and Neosaxitoxin. J. Agric. Food Chem..

[90] McCall J.R., Holland W.C., Keeler D.M., Hardison D.R., Litaker R.W. (2019). Improved Accuracy of Saxitoxin Measurement Using an Optimized Enzyme-Linked Immunosorbent Assay. Toxins.

[91] Harrison K., Johnson S., Turner A.D. (2016). Application of rapid test kits for the determination of paralytic shellfish poisoning (PSP) toxins in bivalve molluscs from Great Britain. Toxicon.

[92] Carmody E.P., James K.J., Kelly S.S. (1995). Diarrhetic Shellfish Poisoning: Evaluation of Enzyme-Linked Immunosorbent Assay Methods for Determination of Dinophyslstoxin-2. J. AOAC Int..

[93] Chin J.D., A Quilliam M., Fremy J.M., Mohapatra S.K., Skorska H.M. (1995). Screening for Okadaic Acid by Immunoassay. J. AOAC Int..

[94] Draisci R., Croci L., Giannetti L., Cozzi L., Lucentini L., De Medici D., Stacchini A. (1994). Comparison of mouse bioassay, HPLC and enzyme immunoassay methods for determining diarrhetic shellfish poisoning toxins in mussels. Toxicon.

[95] Morton S.L., Donald R. (1996). Determination of okadaic acid content of dinoflagellate cells: A comparison of the HPLC-fluorescent method and two monoclonal antibody ELISA test kits. Toxicon.

[96] Tubaro A., Sosa S., Bruno M., Gucci P.M.B., Volterra L., Della Loggia R. (1992). Diarrhoeic shellfish toxins in Adriatic Sea mussels evaluated by an ELISA method. Toxicon.

[97] Matsuura S., Kita H., Takagaki Y. (1994). Specificity of Mouse Monoclonal Anti-Okadaic Acid Antibodies to Okadaic Acid and Its Analogs among Diarrhetic Shellfish Toxins. Biosci. Biotechnol. Biochem..

[98] Suzuki T., Ota H., Yamasaki M. (1999). Direct evidence of transformation of dinophysistoxin-1 to 7-O-acyl-dinophysistoxin-1 (dinophysistoxin-3) in the scallop *Patinopecten yessoensis*. Toxicon.

[99] Johnson S., Harrison K., Turner A.D. (2016). Application of rapid test kits for the determination of Amnesic Shellfish Poisoning in bivalve molluscs from Great Britain. Toxicon.

[100] Johnson S., Harrison K., Turner A.D. (2016). Application of rapid test kits for the determination of Diarrhetic Shellfish Poisoning (DSP) toxins in bivalve molluscs from Great Britain. Toxicon.

[101] Turner A.D., Goya A.B. (2016). Comparison of four rapid test kits for the detection of okadaic acid-group toxins in bivalve shellfish from Argentina. Food Control..

[102] Garthwaite I., Ross K.M., Miles C.O., Hansen R.P., Foster D., Wilkins A.L., Towers N.R. (1998). Polyclonal antibodies to domoic acid, and their use in immunoassays for domoic acid in sea water and shellfish. Nat. Toxins.

[103] Kawatsu K., Hamano Y., Noguchi T. (1999). Production and characterization of a monoclonal antibody against domoic acid and its application to enzyme immunoassay. Toxicon.

[104] Newsome H., Truelove J., Hierlihy L., Collins P. (1991). Determination of domoic acid in serum and urine by immunochemical analysis. Bull. Environ. Contam. Toxicol..

[105] Osada M., Marks L., Stewart J. (1995). Determination of domoic acid by two different versions of a competitive enzyme-linked immunosorbent assay (ELISA). Bull. Environ. Contam. Toxicol..

[106] Rhodes L., Scholin C., Garthwaite I. (1998). Pseudo-nitzschia in New Zealand and the role of DNA probes and immunoassays in refining marine biotoxin monitoring programmes. Nat. Toxins.

[107] Saeed A.F.U.H., Ling S., Yuan J., Wang S. (2017). The Preparation and Identification of a Monoclonal Antibody against Domoic Acid and Establishment of Detection by Indirect Competitive ELISA. Toxins.

[108] Sanchis A., Bosch-Orea C., Salvador J.-P., Marco M.-P., Farré M. (2019). Development and validation of a multianalyte immunoassay for the quantification of environmental pollutants in seawater samples from the Catalonia coastal area. Anal. Bioanal. Chem..

[109] Smith D., Kitts D. (1994). A competitive enzyme-linked immunoassay for domoic acid determination in human body fluids. Food Chem. Toxicol..

[110] Tsao Z.-J., Liao Y.-C., Liu B.-H., Su C.-C., Yu F.-Y. (2007). Development of a Monoclonal Antibody against Domoic Acid and Its Application in Enzyme-Linked Immunosorbent Assay and Colloidal Gold Immunostrip. J. Agric. Food Chem..

[111] Yu F.-Y., Liu B.-H., Wu T.-S., Chi T.-F., Su M.-C. (2004). Development of a Sensitive Enzyme-Linked Immunosorbent Assay for the Determination of Domoic Acid in Shellfish. J. Agric. Food Chem..

[112] Kleivdal H., Kristiansen S.-I., Nilsen M.V., Briggs L. (2007). Single-Laboratory Validation of the Biosense Direct Competitive Enzyme-Linked Immunosorbent Assay (ELISA) for Determination of Domoic Acid Toxins in Shellfish. J. AOAC Int..

[113] Kleivdal H., Kristiansen S.-I., Nilsen M.V., Goksyr A., Briggs L., Holland P., McNabb P., Aasheim A., Aune T., Bates S. (2007). Determination of Domoic Acid Toxins in Shellfish by Biosense ASP ELISAA Direct Competitive Enzyme-Linked Immunosorbent Assay: Collaborative Study. J. AOAC Int..

[114] Shaw I., O’Reilly A., Charleton M., Kane M. (2008). Development of a High-Affinity Anti-Domoic Acid Sheep scFv and its Use in Detection of the Toxin in Shellfish. Anal. Chem..

[115] Ling S., Xiao S., Xie C., Wang R., Zeng L., Wang K., Zhang D., Li X., Wang S. (2018). Preparation of Monoclonal Antibody for Brevetoxin 1 and Development of Ic-ELISA and Colloidal Gold Strip to Detect Brevetoxin 1. Toxins.

[116] Briggs L.R., Miles C.O., Fitzgerald J.M., Ross K.M., Garthwaite I., Towers N.R. (2004). Enzyme-Linked Immunosorbent Assay for the Detection of Yessotoxin and Its Analogues. J. Agric. Food Chem..

[117] Samdal I.A., Løvberg K.E., Briggs L.R., Kilcoyne J., Xu J., Forsyth C.J., Miles C.O. (2015). Development of an ELISA for the Detection of Azaspiracids. J. Agric. Food Chem..

[118] Reverté L., De La Iglesia P., Del Río V., Campbell K., Elliott C.T., Kawatsu K., Katikou P., Diogène J., Campàs M. (2015). Detection of Tetrodotoxins in Puffer Fish by a Self-Assembled Monolayer-Based Immunoassay and Comparison with Surface Plasmon Resonance, LC-MS/MS, and Mouse Bioassay. Anal. Chem..

[119] Reverté L., Rambla-Alegre M., Leonardo S., Bellés C., Campbell K., Elliott C.T., Gerssen A., Klijnstra M.D., Diogène J., Campàs M. (2018). Development and validation of a maleimide-based enzyme-linked immunosorbent assay for the detection of tetrodotoxin in oysters and mussels. Talanta.

[120] Tsumuraya T., Sato T., Hirama M., Fujii I. (2018). Highly Sensitive and Practical Fluorescent Sandwich ELISA for Ciguatoxins. Anal. Chem..

[121] CRLMB (Community Reference Laboratory for Marine Biotoxins) (2005). Report on Toxicology Working Group Meeting. http://www.aesan.msps.es/en/CRLMB/web/home.shtml.

[122] Friedman M.A., Fernandez M., Backer L.C., Dickey R.W., Bernstein J., Schrank K., Kibler S., Stephan W., Gribble M.O., Bienfang P. (2017). An Updated Review of Ciguatera Fish Poisoning: Clinical, Epidemiological, Environmental, and Public Health Management. Mar. Drugs.

[123] U.S. FDA (United States Food and Drug Administration) (2020). Fish and Fishery Products Hazards and Controls Guidance (Fourth). https://www.fda.gov/media/80637/download.

[124] Boscolo S., Pelin M., De Bortoli M., Fontanive G., Barreras A., Berti F., Sosa S., Chaloin O., Bianco A., Yasumoto T. (2013). Sandwich ELISA Assay for the Quantitation of Palytoxin and Its Analogs in Natural Samples. Environ. Sci. Technol..

[125] Jawaid W., Campbell K., Melville K., Holmes S.J., Rice J., Elliott C.T. (2015). Development and Validation of a Novel Lateral Flow Immunoassay (LFIA) for the Rapid Screening of Paralytic Shellfish Toxins (PSTs) from Shellfish Extracts. Anal. Chem..

[126] Dorantes-Aranda J.J., Tan J.Y.C., Hallegraeff G.M., Campbell K., Ugalde S.C., Harwood D.T., Bartlett J.K., Campàs M., Crooks S., Gerssen A. (2018). Detection of Paralytic Shellfish Toxins in Mussels and Oysters Using the Qualitative Neogen Lateral-Flow Immunoassay: An Interlaboratory Study. J. AOAC Int..

[127] Jawaid W., Meneely J., Campbell K., Hooper M., Melville K., Holmes S., Rice J., Elliott C. (2013). Development and validation of the first high performance-lateral flow immunoassay (HP-LFIA) for the rapid screening of domoic acid from shellfish extracts. Talanta.

[128] Jawaid W., Meneely J.P., Campbell K., Melville K., Holmes S.J., Rice J., Elliott C.T. (2015). Development and Validation of a Lateral Flow Immunoassay for the Rapid Screening of Okadaic Acid and All Dinophysis Toxins from Shellfish Extracts. J. Agric. Food Chem..

[129] Laycock M.V., Jellett J.F., Belland E.R., Bishop P.C., Thériault B., Russell-Tattrie A.L., Quilliam M.A., Cembella A.D., Richards R.C. (2001). MIST AlertTM: A Rapid Assay for Paralytic Shellfish Poisoning Toxins. Intergovernmental Panel on Climate Change.

[130] Laycock M.V., Jellett J.F., Easy D.J., Donovan M.A. (2006). First report of a new rapid assay for diarrhetic shellfish poisoning toxins. Harmful Algae.

[131] Turner A.D., Tarnovius S., Johnson S., Higman W.A., Algoet M. (2015). Testing and application of a refined rapid detection method for paralytic shellfish poisoning toxins in UK shellfish. Toxicon.

[132] Shen H., Xu F., Xiao M., Fu Q., Cheng Z., Zhang S., Huang C., Tang Y. (2017). A new lateral-flow immunochromatographic strip combined with quantum dot nanobeads and gold nanoflowers for rapid detection of tetrodotoxin. Analyst.

[133] Nelis J.L.D., Tsagkaris A., Zhao Y., Lou-Franco J., Nolan P., Zhou H., Cao C., Rafferty K., Hajslova J., Elliott C. (2019). The end user sensor tree: An end-user friendly sensor database. Biosens. Bioelectron..

[134] Szkola A., Campbell K., Elliott C.T., Niessner R., Seidel M. (2013). Automated, high performance, flow-through chemiluminescence microarray for the multiplexed detection of phycotoxins. Anal. Chim. Acta.

[135] Fraga M., Vilariño N., Louzao M.C., Rodriguez P., Campbell K., Elliott C.T., Botana L.M. (2013). Multidetection of Paralytic, Diarrheic, and Amnesic Shellfish Toxins by an Inhibition Immunoassay Using a Microsphere-Flow Cytometry System. Anal. Chem..

[136] Rodríguez L.P., Vilariño N., Louzao M.C., Dickerson T.J., Nicolaou K.C., Frederick M.O., Botana L.M. (2014). Microsphere-based immunoassay for the detection of azaspiracids. Anal. Biochem..

[137] Rodríguez L.P., Vilariño N., Molgó J., Araoz R., Louzao M.C., Taylor P., Talley T., Botana L.M. (2013). Development of a Solid-Phase Receptor-Based Assay for the Detection of Cyclic Imines Using a Microsphere-Flow Cytometry System. Anal. Chem..

[138] Campbell K., Stewart L.D., Doucette G.J., Fodey T.L., Haughey S.A., Vilariño N., Kawatsu K., Elliott C.T. (2007). Assessment of Specific Binding Proteins Suitable for the Detection of Paralytic Shellfish Poisons Using Optical Biosensor Technology. Anal. Chem..

[139] Campbell K., Rawn D., Niedzwiadek B., Elliott C. (2011). Paralytic shellfish poisoning (PSP) toxin binders for optical biosensor technology: Problems and possibilities for the future: A review. Food Addit. Contam. Part A.

[140] Campbell K., Barnes P., Haughey S.A., Higgins C., Kawatsu K., Vasconcelos V., Elliott C.T. (2013). Development and single laboratory validation of an optical biosensor assay for tetrodotoxin detection as a tool to combat emerging risks in European seafood. Anal. Bioanal. Chem..

[141] Campbell K., Haughey S.A., Top H.V.D., Van Egmond H., Vilariño N., Botana L.M., Elliott C.T. (2010). Single Laboratory Validation of a Surface Plasmon Resonance Biosensor Screening method for Paralytic Shellfish Poisoning Toxins. Anal. Chem..

[142] Campbell K., Huet A.-C., Charlier C., Higgins C., Delahaut P., Elliott C.T. (2009). Comparison of ELISA and SPR biosensor technology for the detection of paralytic shellfish poisoning toxins. J. Chromatogr. B.

[143] Haughey S.A., Campbell K., Yakes B.J., Prezioso S.M., DeGrasse S.L., Kawatsu K., Elliott C.T. (2011). Comparison of biosensor platforms for surface plasmon resonance based detection of paralytic shellfish toxins. Talanta.

[144] Prado E., Colas F., Laurent S., Tardivel M., Evrard J., Forest B., Boche A., Rouxel J. Toward a SPR imaging in situ system to detect marine biotoxin. Proceedings of the SPIE 11361, Biophotonics in Point-of-Care, 113610J.

[145] Van Den Top H.J., Elliott C.T., Haughey S.A., Vilariño N., Van Egmond H.P., Botana L.M., Campbell K. (2011). Surface Plasmon Resonance Biosensor Screening Method for Paralytic Shellfish Poisoning Toxins: A Pilot Interlaboratory Study. Anal. Chem..

[146] Yakes B.J., DeGrasse S.L., Poli M., Deeds J.R. (2011). Antibody characterization and immunoassays for palytoxin using an SPR biosensor. Anal. Bioanal. Chem..

[147] Yu Q., Chen S., Taylor A.D., Homola J., Hock B., Jiang S. (2005). Detection of low-molecular-weight domoic acid using surface plasmon resonance sensor. Sens. Actuators B Chem..

[148] Fonfría E.S., Vilariño N., Campbell K., Elliott C., Haughey S.A., Ben-Gigirey B., Vieites J.M., Kawatsu A.K., Botana L.M. (2007). Paralytic Shellfish Poisoning Detection by Surface Plasmon Resonance-Based Biosensors in Shellfish Matrixes. Anal. Chem..

[149] Stevens R.C., Soelberg S.D., Eberhart B.-T.L., Spencer S., Wekell J.C., Chinowsky T., Trainer V.L., Furlong C.E. (2007). Detection of the toxin domoic acid from clam extracts using a portable surface plasmon resonance biosensor. Harmful Algae.

[150] Llamas N.M., Stewart L., Fodey T., Higgins H.C., Velasco M.L.R., Botana L.M., Elliott C.T. (2007). Development of a novel immunobiosensor method for the rapid detection of okadaic acid contamination in shellfish extracts. Anal. Bioanal. Chem..

[151] McNamee S.E., Elliott C.T., Delahaut P., Campbell K. (2012). Multiplex biotoxin surface plasmon resonance method for marine biotoxins in algal and seawater samples. Environ. Sci. Pollut. Res..

[152] Campbell K., McNamee S.E., Huet A.-C., Delahaut P., Vilariño N., Botana L.M., Poli M., Elliott C.T. (2014). Evolving to the optoelectronic mouse for phycotoxin analysis in shellfish. Anal. Bioanal. Chem..

[153] Bratakou S., Nikoleli G.-P., Siontorou C.G., Nikolelis D.P., Karapetis S., Tzamtzis N. (2017). Development of an Electrochemical Biosensor for the Rapid Detection of Saxitoxin Based on Air Stable Lipid Films with Incorporated Anti-STX Using Graphene Electrodes. Electroanalysis.

[154] Leonardo S., Kilcoyne J., Samdal I.A., Miles C.O., O’Sullivan C.K., Diogene J., Campàs M. (2018). Detection of azaspiracids in mussels using electrochemical immunosensors for fast screening in monitoring programs. Sens. Actuators B Chem..

[155] Zamolo V.A., Valenti G., Venturelli E., Chaloin O., Marcaccio M., Boscolo S., Castagnola V., Sosa S., Berti F., Fontanive G. (2012). Highly Sensitive Electrochemiluminescent Nanobiosensor for the Detection of Palytoxin. ACS Nano.

[156] McNamee S.E., Elliott C.T., Greer B., Lochhead M., Campbell K. (2014). Development of a Planar Waveguide Microarray for the Monitoring and Early Detection of Five Harmful Algal Toxins in Water and Cultures. Environ. Sci. Technol..

[157] Reverté L., Campàs M., Yakes B.J., Deeds J.R., Katikou P., Kawatsu K., Lochhead M., Elliott C.T., Campbell K. (2017). Tetrodotoxin detection in puffer fish by a sensitive planar waveguide immunosensor. Sens. Actuators B Chem..

[158] Campàs M., Marty J.-L. (2007). Enzyme sensor for the electrochemical detection of the marine toxin okadaic acid. Anal. Chim. Acta.

[159] Zhou J., Qiu X., Su K., Xu G., Wang P. (2016). Disposable poly (o-aminophenol)-carbon nanotubes modified screen print electrode-based enzyme sensor for electrochemical detection of marine toxin okadaic acid. Sens. Actuators B Chem..

[160] Ye W., Liu T., Zhang W., Zhu M., Liu Z., Kong Y., Liu S. (2019). Marine Toxins Detection by Biosensors Based on Aptamers. Toxins.

[161] Gao S., Hu B., Zheng X., Cao Y., Liu D., Sun M., Jiao B., Wang L. (2016). Gonyautoxin 1/4 aptamers with high-affinity and high-specificity: From efficient selection to aptasensor application. Biosens. Bioelectron..

[162] Gao S., Zheng X., Hu B., Sun M., Wu J., Jiao B., Wang L. (2017). Enzyme-linked, aptamer-based, competitive biolayer interferometry biosensor for palytoxin. Biosens. Bioelectron..

[163] Gao S., Zheng X., Wu J. (2017). A biolayer interferometry-based competitive biosensor for rapid and sensitive detection of saxitoxin. Sens. Actuators B Chem..

[164] Chinnappan R., AlZabn R., Fataftah A.K., Alhoshani A., Zourob M. (2020). Probing high-affinity aptamer binding region and development of aptasensor platform for the detection of cylindrospermopsin. Anal. Bioanal. Chem..

[165] Qiang L., Zhang Y., Guo X., Gao Y., Han Y., Sun J., Han L. (2020). A rapid and ultrasensitive colorimetric biosensor based on aptamer functionalized Au nanoparticles for detection of saxitoxin. RSC Adv..

[166] Nelis J.L.D., Tsagkaris A., Dillon M., Hajslova J., Elliott C. (2020). Smartphone-based optical assays in the food safety field. TrAC Trends Anal. Chem..

[167] Fang J., Qiu X., Wan Z., Zou Q., Su K., Hu N., Wang P. (2016). A sensing smartphone and its portable accessory for on-site rapid biochemical detection of marine toxins. Anal. Methods.

[168] Su K., Qiu X., Fang J., Zou Q., Wang P. (2017). An improved efficient biochemical detection method to marine toxins with a smartphone-based portable system—Bionic e-Eye. Sens. Actuators B Chem..

[169] Chinowsky T.M., Soelberg S.D., Baker P., Swanson N.R., Kauffman P., Mactutis A., Grow M.S., Atmar R., Yee S.S., Furlong C.E. (2007). Portable 24-analyte surface plasmon resonance instruments for rapid, versatile biodetection. Biosens. Bioelectron..

[170] Rahimi F., Chatzimichail S., Saifuddin A., Surman A.J., Taylor-Robinson S.D., Salehi-Reyhani A. (2020). A Review of Portable High-Performance Liquid Chromatography: The Future of the Field?. Chromatographia.

[171] Jensen P.A., Dougherty B.V., Moutinho T.J., Papin J.A. (2015). Miniaturized Plate Readers for Low-Cost, High-Throughput Phenotypic Screening. J. Lab. Autom..

[172] Berg B., Cortazar B., Tseng D., Ozkan H., Feng S., Wei Q., Chan R.Y.-L., Burbano J., Farooqui Q., Lewinski M. (2015). Cellphone-Based Hand-Held Microplate Reader for Point-of-Care Testing of Enzyme-Linked Immunosorbent Assays. ACS Nano.

[173] McElhiney J., Drever M., Lawton L.A., Porter A.J. (2002). Rapid Isolation of a Single-Chain Antibody against the Cyanobacterial Toxin Microcystin-LR by Phage Display and Its Use in the Immunoaffinity Concentration of Microcystins from Water. Appl. Environ. Microbiol..

[174] McElhiney J., Lawton L.A., Porter A.J. (2000). Detection and quantification of microcystins (cyanobacterial hepatotoxins) with recombinant antibody fragments isolated from a naÃ¯ve human phage display library. FEMS Microbiol. Lett..

[175] Hara Y., Dong J., Ueda H. (2013). Open-sandwich immunoassay for sensitive and broad-range detection of a shellfish toxin gonyautoxin. Anal. Chim. Acta.

[176] Peltomaa R., Benito-Peña E., Barderas R., Moreno-Bondi M.C. (2019). Phage Display in the Quest for New Selective Recognition Elements for Biosensors. ACS Omega.

[177] Shriver-Lake L.C., Liu J.L., Lee P.A.B., Goldman E.R., Dietrich R., Märtlbauer E., Anderson G.P. (2016). Integrating scFv into xMAP Assays for the Detection of Marine Toxins. Toxins.

[178] Maguire I., Fitzgerald J., Heery B., Nwankire C., O’Kennedy R., Ducrée J., Regan F. (2018). Novel Microfluidic Analytical Sensing Platform for the Simultaneous Detection of Three Algal Toxins in Water. ACS Omega.

[179] Cunha I., Biltes R., Sales M., Vasconcelos V. (2018). Aptamer-Based Biosensors to Detect Aquatic Phycotoxins and Cyanotoxins. Sensors.

[180] Eissa S., Ng A., Siaj M., Tavares A.C., Zourob M. (2013). Selection and Identification of DNA Aptamers against Okadaic Acid for Biosensing Application. Anal. Chem..

[181] Gu H., Duan N., Wu S., Hao L., Xia Y., Ma X., Wang Z. (2016). Graphene oxide-assisted non-immobilized SELEX of okdaic acid aptamer and the analytical application of aptasensor. Sci. Rep..

[182] Handy S.M., Yakes B.J., DeGrasse J.A., Campbell K., Elliott C.T., Kanyuck K.M., DeGrasse S.L. (2013). First report of the use of a saxitoxin–protein conjugate to develop a DNA aptamer to a small molecule toxin. Toxicon.

[183] Nelis J.L.D., Migliorelli D., Jafari S., Generelli S., Lou-Franco J., Salvador J.P., Marco M.P., Cao C., Elliott C.T., Campbell K. (2020). The benefits of carbon black, gold and magnetic nanomaterials for point-of-harvest electrochemical quantification of domoic acid. Mikrochim. Acta.

[184] Nelis J.L.D., Migliorelli D., Mühlebach L., Generelli S., Stewart L., Elliott C.T., Campbell K. (2021). Highly sensitive electrochemical detection of the marine toxins okadaic acid and domoic acid with carbon black modified screen printed electrodes. Talanta.

[185] Gholami M.D., Sonar P., Ayoko G.A., Izake E.L. (2020). A highly sensitive SERS quenching nanosensor for the determination of tumor necrosis factor alpha in blood. Sens. Actuators B Chem..

[186] Lawrence J.E., Cembella A.D., Ross N.W., Wright J. (1998). Cross-reactivity of an anti-okadaic acid antibody to dinophysistoxin-4 (DTX-4), dinophysistoxin-5 (DTX-5), and an okadaic acid diol ester. Toxicon.

[187] Shestowsky W.S., Holmes C., Hu T., Marr J., Wright J., Chin J., Sikorska H. (1993). An Anti-okadaic Acid-Anti-idiotypic Antibody Bearing an Internal Image of Okadaic Acid Inhibits Protein Phosphatase PP1 and PP2A Catalytic Activity. Biochem. Biophys. Res. Commun..

[188] Shestowsky W.S., Quilliam M.A., Sikorska H.M. (1992). An idiotypic-anti-idiotypic competitive immunoassay for quantitation of okadaic acid. Toxicon.

[189] Schulz K., Pöhlmann C., Dietrich R., Märtlbauer E., Elßner T. (2019). Electrochemical Biochip Assays Based on Anti-idiotypic Antibodies for Rapid and Automated On-Site Detection of Low Molecular Weight Toxins. Front. Chem..

[190] Peacock M.B., Gibble C.M., Senn D.B., Cloern J.E., Kudela R.M. (2018). Blurred lines: Multiple freshwater and marine algal toxins at the land-sea interface of San Francisco Bay, California. Harmful Algae.

[191] Hu T., Curtis J.M., Walter J.A., McLachlan J.L., Wright J.L. (1995). Two new water-soluble dsp toxin derivatives from the dinoflagellate prorocentrum maculosum: Possible storage and excretion products. Tetrahedron Lett..

[192] Li A., Chen H., Qiu J., Lin H., Gu H. (2016). Determination of multiple toxins in whelk and clam samples collected from the Chukchi and Bering seas. Toxicon.

[193] Fang L., Yao X., Wang L., Li J. (2015). Solid-Phase Extraction-Based Ultra-Sensitive Detection of Four Lipophilic Marine Biotoxins in Bivalves by High-Performance Liquid Chromatography-Tandem Mass Spectrometry. J. Chromatogr. Sci..

[194] Puech L., Dragacci S., Gleizes E., Fremy J.-M. (1999). Use of immunoaffinity columns for clean-up of diarrhetic toxins (okadaic acid and dinophysistoxins) extracts from shellfish prior to their analysis by HPLC/fluorimetry. Food Addit. Contam..

[195] Chen J., Gao L., Li Z., Wang S., Li J., Cao W., Sun C., Zheng L., Wang X. (2016). Simultaneous screening for lipophilic and hydrophilic toxins in marine harmful algae using a serially coupled reversed-phase and hydrophilic interaction liquid chromatography separation system with high-resolution mass spectrometry. Anal. Chim. Acta.

[196] Devlin R., Campbell K., Kawatsu K., Elliott C. (2011). Studies in the Use of Magnetic Microspheres for Immunoaffinity Extraction of Paralytic Shellfish Poisoning Toxins from Shellfish. Toxins.

[197] Bragg W.A., Garrett A., I Hamelin E., Coleman R.M., Campbell K., Elliott C.T., Johnson R.C. (2018). Quantitation of saxitoxin in human urine using immunocapture extraction and LC–MS. Bioanalysis.

